# Biodegradation Rates of Organic Chemicals in a Subtropical River: From Laboratory to Field

**DOI:** 10.1021/acs.est.5c18446

**Published:** 2026-04-01

**Authors:** Lily M. Weir, Run Tian, Malte Posselt, Jochen F. Mueller, Michael S. McLachlan

**Affiliations:** † Queensland Alliance for Environmental Health Sciences (QAEHS), 534457 The University of Queensland, 20 Cornwall Street, Woolloongabba, Queensland 4102, Australia; ‡ Department of Environmental Science (ACES), 7675 Stockholm University, 106 91 Stockholm, Sweden

**Keywords:** persistence, OECD 309, laboratory-field comparison, micropollutants, aquatic ecosystems

## Abstract

Understanding the attenuation rate of organic chemicals in aquatic environments is essential for assessing their long-term ecological risks and informing chemical management. Biodegradation is a key attenuation pathway for many chemicals, however, measuring biodegradation in situ is inherently complex, resource-intensive, and subject to high environmental variability. Consequently, regulatory frameworks and research efforts often rely on laboratory tests, intended to provide controlled and reproducible conditions. Despite their widespread application, few studies have evaluated how well these laboratory results represent biodegradation in real aquatic environments. In this study, we compared biodegradation rate constants (*k*) from a modified OECD 309 laboratory test with those derived from a field experiment in a subtropical river (Queensland, Australia). Biodegradation was measurable in both laboratory and field experiments for 23 chemicals spanning diverse structures. Rank-order agreement between laboratory *k* and field *k* was strong (Spearman *ρ* = 0.7, *p* < 0.001), and linear association was high (Pearson *r* = 0.8, *p* < 0.001) for the 16 chemicals that did not sorb strongly. The relative magnitudes of laboratory and field log *k* also aligned well (slope of the linear regression = 0.8), however, uncertainty remained (average root-mean-square-error equivalent to a factor of 2). These results demonstrate that the modified OECD 309 can capture much of the rank-order and relative magnitude of biodegradation, supporting its role as a higher-tier laboratory simulation test for persistence assessment.

## Graphical abstract

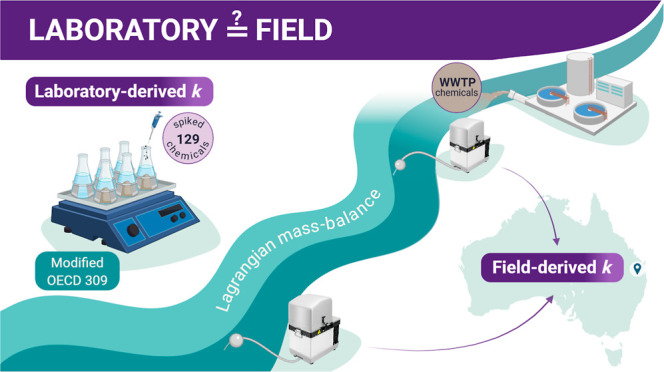

## Introduction

The reversibility of environmental exposure, defined as the capacity of environmental concentrations to decline toward former lower levels after a reduction in emissions, is a key determinant of chemical exposure hazard and of a chemical’s likelihood to be a planetary boundary threat.^1^ Chemical persistence has a strong influence on both the reversibility as well as the magnitude of environmental exposure. The inherent complexity of natural systems has led to the widespread use of controlled laboratory experiments to assess the persistence of chemicals, and these are central to current chemical regulation procedures.^2,3^ Given that biodegradation is a dominant removal pathway for many chemicals,^4–6^ higher-tier chemical persistence assessment commonly relies on laboratory-based biodegradation simulations. Under the REACH (Registration, Evaluation, Authorisation, and Restriction of Chemicals) framework, for example, the OECD 309 protocol (*Aerobic mineralization in surface water* – *simulation biodegradation test* ^7^) is recommended as the first test to support higher-tier persistence assessment.

A premise of simulation tests, such as OECD 309, is that they provide environmentally relevant biodegradation rates. Questions are being raised regarding the OECD 309 test’s relevance to natural aquatic systems as well as its repeatability.^8–11^ We and others have adapted the OECD 309 protocol in an attempt to address these concerns.^8,9^ Perhaps the most important modification was to derive the rate constant from the initial slope of the chemical attenuation curve rather than the maximum slope observed during the ∼10 day simulation, as any change in the slope is an indication of a deviation of the system from its initial (environmental) state.^8,12–14^ Other changes to the protocol included limiting chemical spiking to low concentrations (1 μg L^–1^), spiking multiple chemicals simultaneously, increasing the sediment concentration (and thereby microbial biomass), and reducing the test length to ∼10 days, which was adequate to characterize the initial kinetics while reducing the experimental costs.^8^ Increasing sediment concentration has been reported to enhance the repeatability of OECD 309 simulations,^9,15^ while reducing the spiking concentration decreases the likelihood of the test chemical itself influencing the degrading properties of the test system.^8^

Despite these efforts to improve the environmental relevance of the OECD 309 test, it remains unclear how the rate constants measured in the test relate to biodegradation rate constants in the natural aquatic system that was sampled. Few studies have experimentally evaluated the environmental relevance of laboratory-based biodegradation experiments. Radke and Maier^16^ compared the dissipation half-lives of eight pharmaceuticals in modified OECD 308 laboratory tests with those derived from mass balance modeling in two German rivers. Honti et al.^17^ employed a large-scale catchment modeling approach to estimate biodegradation rate constants from field data for four pharmaceutical active ingredients in the Rhine River basin. They compared these to biodegradation rate constants from standard OECD 308 laboratory tests. A central feature of their methodology was the use of the *k’*_*bio*_ concept–a second-order rate constant normalized to a biomass proxy–which facilitates comparison of field and laboratory-derived rate constants.^17,18^ Seller et al.^2^ applied this *k*’_*bio*_ concept to compare biomass-normalized biodegradation rate constants derived using modified OECD 308 laboratory experiments with those derived using a catchment-scale water quality model of field data for 27 organic chemicals in the Rhine River basin. However, as reported by Honti et al.^18^ and by Shrestha et al.,^19^ OECD 308-style incubations yield system-dependent persistence indicators contingent on vessel geometry (i.e., test setup), sorption-modulated phase transfer, and redox layering, leading to variable outcomes.

In comparison, the OECD 309 test is less susceptible to experimental parameters affecting the test outcome. However, the suspension of the sediment in the OECD 309 incubation represents a significant disruption of the conditions in the field, eliminating redox gradients and mass transfer limitations that exist in sediment deposits. Thus, while the OECD 309 test may be more repeatable, there are significant uncertainties regarding how well it simulates environmental systems. To the best of our knowledge, Li and McLachlan^20^ conducted the only published comparison of biodegradation half-lives from an OECD 309 experiment against half-lives measured in the field. The study was conducted for a hypereutrophic lake receiving wastewater treatment plant (WWTP) effluent in Sweden, and no sediment was added given the very high levels of suspended organic matter already present in the sampled water. They reported that laboratory and field half-lives for 14 of 18 chemicals agreed within a factor of 3, suggesting that the laboratory test did a reasonable job at simulating biodegradation in the field for this hypereutrophic lake. However, this does not address the uncertainty in environmental relevance that arises when sediment is added to the laboratory incubation (or when biodegradation in sediment has a major influence on the overall elimination of chemical from the studied aquatic system).

Against this scientific background, the aim of this study was to assess the efficacy of a modified OECD 309 laboratory experiment in estimating the biodegradation rate constants of a range of organic micropollutants. We tested two hypotheses: (H1) the relative magnitude of chemical biodegradation rate constants is preserved between the laboratory experiment and the field, i.e., chemicals with lower biodegradation rate constants in the laboratory exhibit lower rate constants in the field; (H2) biodegradation rate constants in the field can be predicted from laboratory-derived rate constants using a single proportionality (scaling) factor. This second hypothesis implies a linear relationship between laboratory and field such that once the scaling factor is established using one reference chemical, it can be applied to other chemicals without recalibration.

To test these hypotheses, we conducted an in situ mass-balance experiment in a subtropical Australian river for hydrophilic organic chemicals emitted by a WWTP, and in parallel performed a modified OECD 309 type experiment^8^ with water and sediment collected from the river stretch during the same month. The hypotheses were evaluated by comparing the rate constants obtained from the two experiments.

## Theoretical Framework

Here we present a theoretical framework to facilitate the comparison of the results of the laboratory and field studies of biodegradation in a river. The attenuation of chemicals along a river stretch can be described using a Lagrangian steady-state modeling framework. We write a mass balance for a parcel of water traveling from site A to B while making the following assumptions:

i. There are no inputs of chemical to the water parcel;
ii. The change of volume of the water in the parcel is negligible (i.e., no dilution);
iii. As it travels, the water parcel interacts with a sediment compartment, and the volume and biodegradation properties of this sediment compartment remain constant;
iv. There is equilibrium partitioning of the chemical between water and sediment;
v. Physical loss of chemical from the parcel (e.g., through volatilization or groundwater infiltration) is negligible;
vi. Biodegradation of the chemical can be described as a second order process with respect to the concentration of the chemical in the dissolved phase and the biomass density in the water/sediment system;
vii. Advection dominates dispersion over the travel time (plug flow).

Under these conditions, the mass balance equation for the parcel can be written as

1 $$V_{W}\left(v_{W}+v_{S}K_{SW}\right)\frac{dC_{W}}{dt}=-k_{b}BV_{W}C_{W}$$

where *V*_*W*_ (m^3^) is the volume of water in the parcel and associated sediment, *v*_*W*_ (m^3^ m^–3^) is the volume fraction of water, *v*_*S*_ (m^3^ m^–3^) is the volume fraction of sediment associated with the water (*v*_*W*_ + *v*_*S*_ = 1), *K*_*SW*_ (m^3^ m^–3^) is the sediment:water equilibrium distribution coefficient, *C*_*W*_ (mol m^–3^) is the dissolved concentration of the chemical in the water, *k*_*b*_ (h^–1^ m^3^ g^–1^) is the second order attenuation constant for biodegradation in the river,^18^ and *B* is the biomass density (g m^–3^). After eliminating *V*_*W*_, eq 1 can be integrated and solved for the pseudo first-order rate constant for biodegradation *k* (h^–1^) using measured *C*_*W*_.

2 $$k=k_{b}\frac{B}{\left(v_{W}+v_{S}K_{SW}\right)}=-\frac{\ln\left(\frac{C_{W}}{C_{W0}}\right)}{\Delta t}$$

where *ln*(*C*_*W*_/*C*_*W0*_)/Δ*t* is the slope of the semilogarithmic plot of *C*_*W*_ versus time *t* (h) from the experiment.

If the laboratory experiment is successful at simulating the biodegradation in the field, then *k*_*b*_ will be the same in the field and the laboratory. In this case we can write

3 $$\frac{k_{lab,corr}\left(v_{W,field}+v_{S,field}K_{SW}\right)}{B_{field}}=\frac{k_{lab}\left(v_{W,lab}+v_{S,lab}K_{SW}\right)}{B_{lab}}$$

whereby we relabel *k*_*field*_ to *k*_*lab,corr*_ to indicate that it is now an estimated value based on the assumption that *k*_*b*_ is the same in the field and the laboratory. Rearranging yields an expression for *k*_*lab,corr*_ (as a function of *k*_*lab*_ and three relevant system properties, *B*, *v*_*W*_ and *v*_*S*_).

4 $$k_{lab,corr}=k_{lab}\frac{B_{field}}{B_{lab}}\frac{\left(v_{W,lab}+v_{S,lab}K_{SW}\right)}{\left(v_{W,field}+v_{S,field}K_{SW}\right)}$$

We now have an equation to predict *k*_*field*_ (i.e., *k*_*lab,corr*_) from *k*_*lab*_, whereby we need to know the ratios *B*_*field*_/*B*_*lab*_ and (*v*_*W,lab*_ + *v*_*S,lab*_ *K*_*SW*_)/(*v*_*W,field*_ + *v*_*S,field*_ *K*_*SW*_). *v*_*W,lab*_ and *v*_*W,field*_ are ∼1, while *B*_*field*_/*B*_*lab*_ and (*v*_*S,lab*_ *K*_*SW*_)/(*v*_*S,field*_ *K*_*SW*_) are constants, i.e., independent of the properties of the chemicals, and thus values estimated for one test chemical can be applied to other test chemicals. From eq 4, for nonsorbing chemicals *B*_*field*_/*B*_*lab*_ equals *k*_*lab,corr*_/*k*_*lab*_, so *B*_*field*_/*B*_*lab*_ can be estimated from *k*_*field*_/*k*_*lab*_ for a nonsorbing chemical (see SI, S7.5 for a conceptual evaluation of this relationship). Furthermore, since the active biomass in both the field and the laboratory simulations is expected to be proportional to the amount of sediment, *B*_*field*_/*B*_*lab*_ can be assumed to equal *v*_*s,field*_/*v*_*s,lab*_, which leaves just one scaling factor needed to relate *k*_*lab*_ to *k*_*field*_ for different chemicals.

We note that this interpretation is a consequence of the hypothesis underlying the framework, namely that *k*_*b*_ is the same in the laboratory test as in the field. Transferring sediments from the field, where they can have both anaerobic and aerobic domains with complex redox gradients, to a laboratory environment that is completely aerobic, can alter *k*_*b*_. In this case, *B*_*field*_ would not reflect the biomass density in the field, but rather the equivalent biomass density assuming that the biomass in the field had the same *k*_*b*_ as the biomass in the laboratory experiment.

If, instead of estimating the rate constant *k*, one is interested in estimating the die-away of a chemical along a stretch of river, then one must convert the rate constant to a spatial die-away constant *k*_*d*_ (m^–1^) by dividing *k* by the average linear velocity of the chemical down the river (*U*, m h^–1^). *U* can be approximated as the average of the velocity of the water (*U*_*W*_) and the sorbent (*U*_*S*_), weighted according to the fraction of the chemical associated with each phase.

5 $$U=\frac{U_{W}v_{W,field}+U_{S}v_{S,field}K_{SW}}{\left(v_{W,field}+v_{S,field}K_{SW}\right)}$$

Assuming that the sorbed fraction is associated with bottom sediment and that this has negligible *U*_*S*_, *k*_*d*_ can be approximated from the results of the laboratory study by

6 $$k_{d}=\frac{k_{lab,corr}}{U}=k_{lab}\frac{B_{field}}{B_{lab}}\frac{\left(v_{W,lab}+v_{S,lab}K_{SW}\right)}{U_{W}v_{W,field}}$$

It is interesting to compare eq 4 and eq 6 for strongly sorbing chemicals. For the rate constant *k*, the effects of chemical sorption and biomass density on the laboratory to field extrapolation cancel each other out, resulting in *k*_*lab,corr*_ ≈ *k*_*lab*_. However, for the die-away constant *k*_*d*_, the laboratory to field extrapolation is strongly dependent on the properties of the chemical, with the quotient of *k*_*d*_ and *k*_*lab*_ being proportional to *K*_*SW*_.

Another consideration is differences in pH between the field and laboratory systems. For dissociating chemicals, only the neutral form can as a rule readily cross membranes, and hence pH can influence the intracellular concentration in degrading microorganisms, which in turn can influence the observed rate constant.^21^ To account for this, *k*_*lab*_ can be corrected based on the fraction neutral (*f*_*N*_) expected under field conditions (see SI, S7.2).

## Materials and Methods

### Test Chemicals

A mixture of 129 chemicals was targeted as discussed and published elsewhere.^22^ They included pharmaceuticals, personal care products, drugs, and industrial chemicals, and were selected based on: (i) measured previously in wastewater and surface water, (ii) nonvolatile, (iii) minimally sorbing (log *D*_*ow*_ < 3 at pH 7.3 for 80% of chemicals), and (iv) quantifiable in water with the available analytical methods. Additional information on the test chemicals is available in the SI, S1.

### Site

The selection of the river segment was guided by criteria for in situ assessment of chemical attenuation^23,24^ and the assumptions made in the theoretical framework:

i. A constant, dominant chemical source (i.e., WWTP effluent),
ii. High effluent-to-streamflow ratio (to facilitate chemical quantification), and
iii. Sufficient water residence time in the river section to observe attenuation.

Gowrie Creek is an urban-agricultural waterway of the Condamine catchment in Toowoomba (Queensland, Australia). Our study reach encompassed a 35 km approximate middle thread distance (AMTD) of Gowrie Creek, beginning 0.5 km downstream of the Wetalla WWTP effluent discharge and ending at a downstream gauging station prior to the waterway’s confluence with Westbrook and Oakey Creeks (see Figure 1). The streambed slope over this stretch was approximately 0.5%, and land use in the corridor was primarily agricultural. Effluent from the WWTP is the primary source of flow during dry weather. The technical standard of the WWTP is biological nutrient removal.^25^

**Figure 1:**
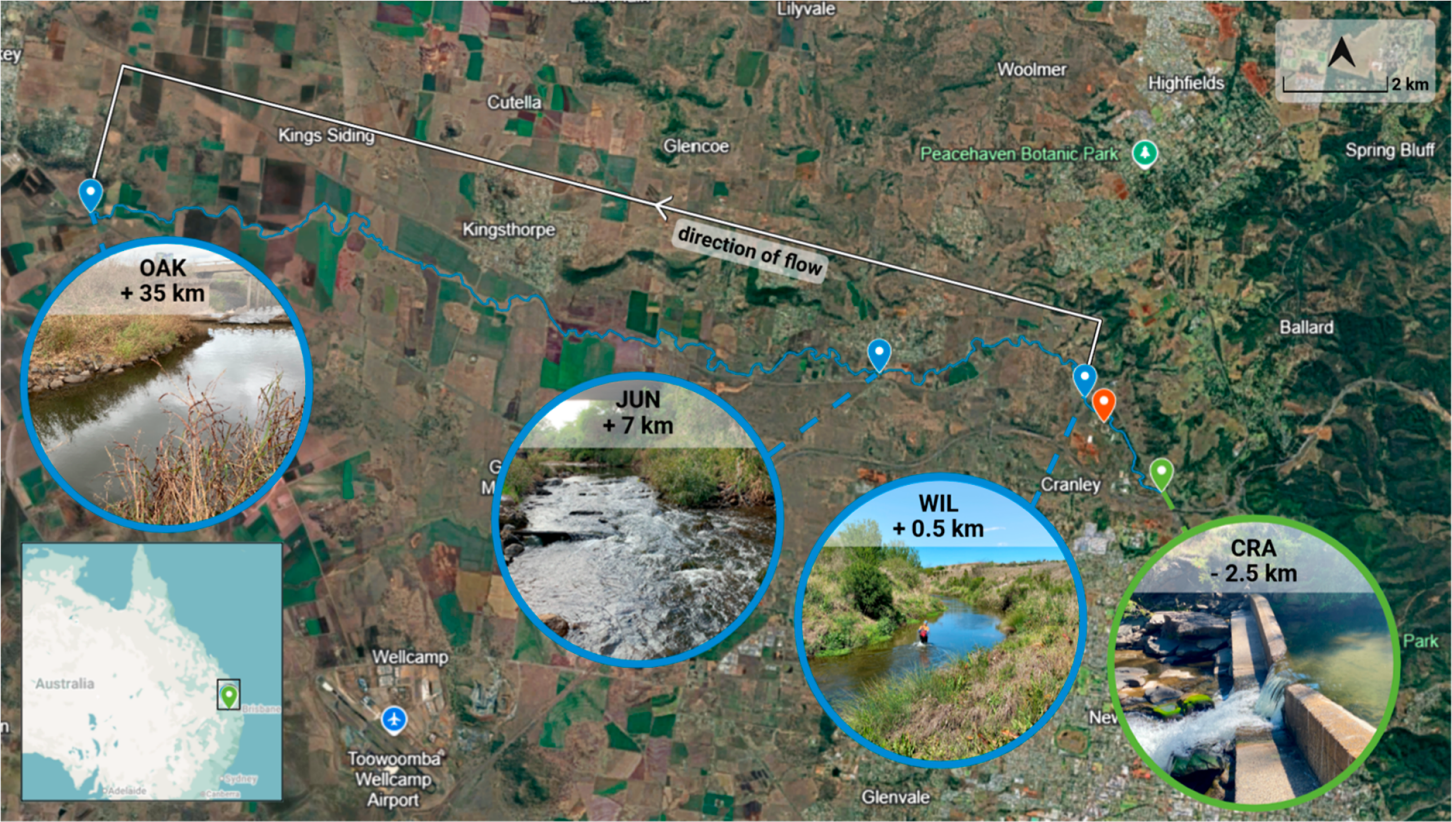
Map of experimental site. Autosamplers were deployed at WIL (+0.5 km relative to WWTP effluent discharge) and OAK (+35 km) for the field experiment. Samples for the laboratory experiment were collected from the sites highlighted blue, while the site used for understanding the baseflow of the system is highlighted green (CRA), and the WWTP effluent location is highlighted red. Map produced using Google Maps (2025) and site attributes overlaid using BioRender (Weir, L.M., 2025, https://BioRender.com/3knc9sv).

Preliminary passive and grab-samples collected prior to the main sampling campaign at multiple locations above and along the study reach confirmed that the WWTP was the dominant source of chemicals to the waterway during dry weather.

Another key consideration was the hydraulic stability of the selected reach as it influences the uncertainty in the mass balance estimates. This was evaluated using discharge data from gauging stations located upstream and downstream of the wastewater treatment plant (WWTP), and effluent discharge data from the WWTP. Daily average discharge was used to characterize between-site dilution and day-to-day variability. At subdaily resolution, the WWTP effluent imposed a pronounced diurnal signal on the flow. This signal was used as a hydraulic tracer to estimate water residence times and to guide the temporal alignment of the upstream and downstream samples (see SI, S2).

### Laboratory Experiment

Laboratory-derived biodegradation rate constants (*k*_*lab*_) were determined using a modified OECD 309 experimental setup and have been published elsewhere.^22^ This experiment quantified initial biodegradation kinetics under oxic conditions in a water/sediment slurry. In short, sediment and surface water were collected from three locations along Gowrie Creek during the same month as the field sampling campaign (see below) and homogenized. The test flasks (triplicate) and sterilized sediment control flasks (duplicate) had a sediment concentration of 50 g of wet solids L^–1^. This sediment concentration exceeds the maximum concentration prescribed in the OECD 309 protocol by a factor of 50. A sterilized sediment-free control flask was also included to assess abiotic transformation processes (hereafter referred to as the hydrolysis control flask). All flasks were spiked with the mixture of 129 chemicals, each at 1 μg L^–1^ (see Table S1) and sealed with Parafilm. The sediment was kept in suspension using an orbital shaker. Incubations were conducted in the dark, in a temperature-controlled room which maintained the ambient water temperature of Gowrie Creek at the time of sampling (19.4 °C). Aliquots were taken from all incubations over a 10 day period and frozen (−20 °C) until analyzed. Physicochemical parameters, including dissolved oxygen, were measured during aliquot sampling (see Weir et al.^22^ for full experimental details, and SI, S7.3 for additional discussion of the treatment of *k*_*lab*_ data).

### Field Experiment

Autosamplers (ISCO Avalanche, Teledyne ISCO) were installed at two sites: one 0.5 km downstream of the WWTP effluent discharge location, and the other 35 km AMTD downstream of the first site. The first site, WIL, had a wetted channel cross-sectional area of 3 m^2^ and a free surface width of 9 m. The second site, OAK, had a wetted channel cross-sectional area of 8 m^2^ and a free surface width of 14 m. The autosamplers were positioned to collect surface water (0.2 m deep) from the middle of the creek channel and were programmed for time-proportional sampling (30 mL of surface water collected every 10 min for 4 h) to fill polyethylene bottles. The offset between autosampler deployment at the two sites was 24 h, based on preliminary data available at the time of deployment that the water travel time between the sampling points was 24 h. Sampling occurred over a period of 24 h in May 2023 which produced six samples at each site. The samples were kept cool inside the autosamplers using ice bricks and, at the completion of the experiment, samples were frozen (−20 °C) until analyzed.

### Water Residence Time

We estimated the water residence time (*τ*) by tracking the time-lagged diurnal signal in specific conductivity (SpCond) and water depth from the WWTP effluent at three locations along Gowrie Creek (WIL, JUN, and OAK, Figure 1). SpCond behaves as a conservative solute tracer and reflects advective transport of the water parcel, whereas fluctuations in water depth represent propagation of a hydraulic pressure wave, which typically travels faster than solute advective transport. These signals were recorded semicontinuously using YSI 6-series sondes (Xylem Water Solutions, Brisbane) and used to constrain water residence times within a one-dimensional reactive transport model in which advection time was treated as the primary calibration parameter.^26,27^ Implicit in this method is the assumption that the WWTP-generated signal is transmitted downstream in a coherent form (i.e., advection dominates, and dispersion and attenuation do not erase the diurnal waveform).

Over the first reach (WIL to JUN), the residence time inferred from the SpCond signal was comparable to the travel time inferred from depth-wave propagation, indicating similar effective velocities at this spatial scale. Over the longer second reach (JUN to OAK), the SpCond signal was too attenuated to permit model fitting, whereas the depth signal remained coherent. The travel time for this reach was therefore inferred from the depth signal. Given that the second reach is substantially longer than the first reach and that the SpCond signal was strongly attenuated at OAK, the travel time derived from the depth signal could exceed the true advective residence time, unlike in the upstream reach where both signals yielded comparable results. Accordingly, this estimate was interpreted as a lower-bound on the water residence time. Using both sets of data, the water residence time from WIL to OAK was estimated at 47 ± 3 h.

### Chemical Analysis

Chemical analysis for both field and laboratory experiments was conducted using ultrahigh-performance liquid chromatography coupled to a Q Exactive HF Hybrid Quadrupole-Orbitrap mass spectrometer (UHPLC-Orbitrap-MS/MS) with electrospray ionization (ESI) as described in previous work.^8^ Details are provided in the SI, S3.

### Laboratory Experiment Biodegradation Rate Constant Calculation

First-order biodegradation rate constants (*k*_*lab*_) were determined using the data from all three test flasks (i.e., replicates) simultaneously with the help of a Python algorithm (chowclassifier)^28^ which included breakpoint identification in the case of biphasic kinetics. When biphasic kinetics were observed, the initial biodegradation rate constant was used. Biodegradation rate constants were deemed “valid” if they were significantly different from zero (*t*-test on the linear regression slope, *p* < 0.05). Chemicals with notable dissipation in the hydrolysis control flask (<2.5 times slower than in the test flasks) were not considered further.

### Field Experiment Biodegradation Rate Constant Calculation

The 24 h average chemical signal at the two sites (*A*_*WIL*_ and *A*_*OAK*_) was determined by averaging the signal intensity in the six 4 h composite samples following outlier detection and removal (based on the interquartile range for each chemical and site). *A*_*OAK*_ was corrected for dilution using a dilution factor (*df* = 1.1, i.e., 10% dilution) derived from discharge data gathered upstream of the WWTP outfall (at CRA gauge), from the WWTP outfall (effluent discharge data), and at the downstream site (at OAK gauge). This dilution estimate was cross-validated using conservative WWTP-derived tracers (PFAS), which produced comparable results (see SI S2.3). First-order attenuation rate constants (*k*_*field*_) were calculated using the dilution-corrected downstream signal, the distance between sites (*d*, m), and the average linear velocity of the chemical between sites (*U*, m h^–1^), as per eq 7.

7 $$k_{field}=\ln\left(\frac{{\mathrm{A̅}}_{WIL}}{{\mathrm{A̅}}_{OAK}\times df}\right)\frac{U}{d}$$

The chemical velocity *U* was calculated using eq 5 as a phase-weighted average of the water velocity and sorbent velocity. Because the field-specific sediment association term (*v*_*S,field*_ *K*_*SW*_) was not directly measured, this was inferred from the laboratory experiment and adjusted to field conditions using the biomass-density scaling factor *B*_*field*_ */B*_*lab*_. Uncertainty in the estimation of *k*_*field*_ was quantified through error propagation of log-transformed peak area variability at the upstream and downstream sites (see SI, S5.2). Confidence intervals (95%) were constructed using a normal approximation (i.e., a z-based 95% interval). Compounds showing significant temporal trends (Kendall’s *τ*, *p* < 0.05) were flagged due to the potential for such trends to bias rate constant estimates. Rate constants were processed for both directly injected and SPE-concentrated samples, and *k*_*field*_ derived from directly injected samples was prioritised over that from SPE-concentrated samples, as direct injection was less impacted by matrix-interferences (see SI, S4 for a comparison of DI and SPE results).

In order to maximize the use of available data while accounting for varying levels of reliability, *k*_*field*_ was categorized into four tiers. Observations with *A* < LOQ, were excluded from the analysis. Tier (i) included chemicals with *A*_*OAK*_ significantly lower than *A*_*WIL*_ (*t*-test, *p* < 0.05) and no significant temporal trend at either site (Kendall’s *τ*, *p* > 0.05). Tier (ii) chemicals were similar to tier (i) chemicals except that the difference between *A*_*OAK*_ and *A*_*WIL*_ was not significant (tier (ii*a*)), or significant temporal variability was observed at one or both sites (tier (ii*b*)). Tier (iii) included chemicals with *A*_*OAK*_ > LOQ for only one or two of the six samples. Tier (iv) comprised chemicals where the downstream site signal was substituted with the LOQ due to all replicates being below the LOQ.

We sought to isolate biodegradation by screening out chemicals for which abiotic losses were potentially dominant. Photodegradation was qualitatively assessed from existing studies; in particular, Paganoni^29^ employed a suite of organic chemicals and an analytical workflow comparable to the current study, providing directly relevant photodegradation data for many analytes. These results were supplemented with a wider literature review to establish which chemicals were consistently reported as photolabile (see SI, S7; Table S6). Where the majority of literature sources agreed, chemicals were classified as either photodegrading (“Yes”) or not (“No”); where evidence was mixed or inconclusive, the classification was marked as “Uncertain”. Chemicals assigned “No” or “Uncertain” were retained and assumed to primarily undergo biodegradation.

### Hypothesis Testing

The hypotheses were tested by comparing *k*_*field*_ and *k*_*lab,corr*_ *. k*_*lab,corr*_ was calculated from *k*_*lab*_ according to eq 4 after correcting *k*_*lab*_ for the difference in pH between the laboratory and the field (see SI, S7.2). All rate constants were log transformed to improve the normality of the data sets. H1 was tested with Spearman’s correlation (*ρ*) to evaluate the rank order concordance. For H2, linear covariation was then assessed using Pearson’s correlation (*r*). The hypothesized proportional scaling between laboratory and field rate constants was evaluated using ordinary least-squares (linear regression) of log *k*_*lab,corr*_ on log *k*_*field*_, with inference focused on the proximity of the slope (*m*) to 1. Overall agreement was summarized using the root-mean-square error (RMSE) between log *k*_*field*_ and log *k*_*lab,corr*_ and reported as a fold difference on a linear scale for ease of interpretation.

## Results and Discussion

### Laboratory Results

Of the 129 chemicals spiked, 104 were detected and 62 had a valid *k*_*lab*_ (significant degradation, *p* < 0.05). The relative standard deviations in *k*_*lab*_ for the three replicate flasks was <30% for 92% of the chemicals. This level of reproducibility was consistent with results from previous studies applying the same method.^21–23^

### Field Results

The daily average discharge data indicated baseflow conditions and limited variability at the daily time scale.

Of the 129 chemicals spiked in the laboratory experiment, 84 were detected in the field experiment. Three of these were below the LOQ (for both the direct injection and SPE-concentrated methods of analysis) and thus excluded. Of the remaining analytes, 60 demonstrated a loss trend (i.e., *A*_*OAK*_ × *df* < *A*_*WIL*_). Of these, 16 were excluded because photodegradation was judged to potentially contribute significantly to in-stream attenuation (see SI, S7.4 for discussion regarding photodegrading chemicals). Of the remaining 44 chemicals for which *k*_*field*_ was estimated, 23 were classified as tier (i), 15 were classified as tier (ii*a*), two were classified as tier (ii*b*), and four were classified as tier (iii). See SI Data set S2.

To contextualize the field results, log-transformed rate constants from this study were compared with the average log-transformed rate constants from other published field studies after correction for key environmental differences. Agreement was variable among chemicals: while several chemicals fell within the same order of magnitude as reported values, literature rate constants were frequently higher than those observed here (see SI, S6 and Data set S1). Such variability is consistent with the wide range of biodegradation rate constants between sites reported from laboratory tests.^21,22,30^

Of the 44 chemicals with valid *k*_*field*_, 23 overlapped with the laboratory data. The laboratory data also provided information on chemical partitioning, expressed as the fraction of chemical present in the dissolved phase (*f*_*dis*_; 0–1, with lower values indicating stronger sorption). Based on *f*_*dis*_, 6 of the 23 chemicals demonstrated no sorption (*f*_*dis*_ ≥ 0.95), 7 demonstrated weak sorption (0.8 > *f*_*dis*_ < 0.95), 3 demonstrated moderate sorption (0.5 ≥ *f*_*dis*_ ≤ 0.8), and 7 demonstrated strong sorption (*f*_*dis*_ < 0.5). *f*_*dis*_ is equal to the quotient of *v*_*W*_ and (*v*_*W*_ + *v*_*S*_ *K*_*SW*_), and it can be converted to field conditions using the biomass densities.

### Comparison of Field and Laboratory *k*

To determine the field-to-laboratory biomass density ratio (*B*_*field*_ */B*_*lab*_), we used the log-transformed rate constants for the six nonsorbing chemicals (*f*_*dis*_ ≥ 0.95), as for such chemicals *B*_*field*_ */B*_*lab*_ is equal to *k*_*field*_/*k*_*lab*_ (see Theoretical Framework). The average biomass ratio was 0.98, indicating that log *k*_*field*_ was, on average, 2% lower than log *k*_*lab*_. This suggests that microbial activity per unit volume was approximately equal in both systems. Given that previous studies have suggested that laboratory-derived biodegradation rate constants require scaling to account for differences in biomass density between laboratory systems and the environment,^2,20^ this outcome was not expected. It suggests that, under the environmental conditions examined here, the biomass concentration chosen for the laboratory experiment (50 g of wet solids L^–1^) fortuitously gave a good representation of the average biomass density in the studied segment of Gowrie Creek. With an average water depth in Gowrie Creek of ∼0.5 m and assuming a wet solids content of the surface sediment of 1200 g L^–1^,^31^ a *B*_*field*_ of 49 g L^–1^ corresponds to an average surface sediment depth of ∼2 cm. For comparison, the depth of the oxygenated surface sediment layer in shallow flowing rivers typically lies in the range of 0–5 cm,^32–34^ which indicates that our measured *B*_*field*_ is consistent with the expected quantity of biomass present in the oxygenated portion of Gowrie Creek. In addition, the average turbidity of Gowrie Creek during the study period was ∼6 NTU, corresponding to a suspended solids concentration of ∼3 mg L^–1^. Even allowing for uncertainty in this estimate, the suspended sediment concentration is orders of magnitude lower than the biomass density, indicating that the dominant source of biomass in the field system resided in the benthic surface sediment rather than the suspended load. This supports our assumption that the sorbed fraction had negligible transport velocity relative to the water phase (*U*_*S*_ = 0). We note that errors in the travel time would impact the biomass ratio estimate, since *k*_*field*_ is inversely proportional to the travel time.

The pH corrected *k*_*lab*_ results (see SI, S7.2 for details regarding this correction) and the average biomass density ratio (i.e., 0.98) were then combined with chemical-specific *f*_*dis*_ to derive *k*_*lab,corr*_ for all chemicals according to eq 4. *k*_*field*_ and *k*_*lab,corr*_ were compared for all 23 chemicals as shown in Figure 2. The majority of chemicals showed good agreement between *k*_*field*_ and *k*_*lab,corr*_, but seven chemicals deviated significantly from a 1:1 relationship (chemicals whose 95% confidence intervals did not intersect the 1:1 line; highlighted red in Figure 2), with *k*_*lab,corr*_ substantially exceeding *k*_*field*_ (except for one chemical showing lower *k*_*lab,corr*_). These deviations were predominantly associated with strongly sorbing chemicals: five of the seven chemicals classified as strongly sorbing (dark blue markers in Figure 2) showed *k*_*lab,corr*_ more than 3-fold higher than *k*_*field*_. Quantitative measures of agreement were therefore evaluated separately for the strongly sorbing chemicals. For the subset of 16 nonsorbing to moderately sorbing chemicals, *k*_*field*_ and *k*_*lab,corr*_ were strongly associated (Pearson *r* = 0.8, and Spearman *ρ* = 0.7; both *p* < 0.001, Figure 2), with a regression slope close to unity (*m* = 0.8). Deviations from a 1:1 relationship were moderate (RMSE of 0.3 log units, or a factor of 2), with *k*_*lab,corr*_ falling within a factor of 2 of *k*_*field*_ for 12 chemicals (and within a factor of 3 for 14 chemicals). Chemicals falling outside the factor-of-two bounds (see red dashed lines in Figure 2) that were not classified as outliers, were a mix of nonsorbing or weakly sorbing chemicals that exhibited high uncertainty (95% CI intersecting the 1:1 line). Overall, the regression line fell close to and intersected the 1:1 line, indicating little systematic difference between *k*_*field*_ and *k*_*lab,corr*_ for nonsorbing to moderately sorbing chemicals. In contrast, strongly sorbing chemicals exhibited a significant positive bias relative to a 1:1 relationship (one-sample *t*-test, *p* < 0.05), with *k*_*lab,corr*_ averaging approximately 5-fold higher than *k*_*field*_, indicating a systematic offset between *k*_*lab,corr*_ and *k*_*field*_ (see SI, S7.1). A key assumption in the theoretical framework is that *v*_*S,field*_/*v*_*s,lab*_ equals *B*_*field*_/*B*_*lab*_. This requires either a homogeneous distribution of active biodegrading organisms and sorption sites in the sediment (which is unlikely), or that sediment sampling captures comparable portions of sediment with high biodegrading capacity and high sorption capacity. If this is not the case, the assumption would not be valid, which would explain the different behavior of the strongly sorbing chemicals.

**Figure 2:**
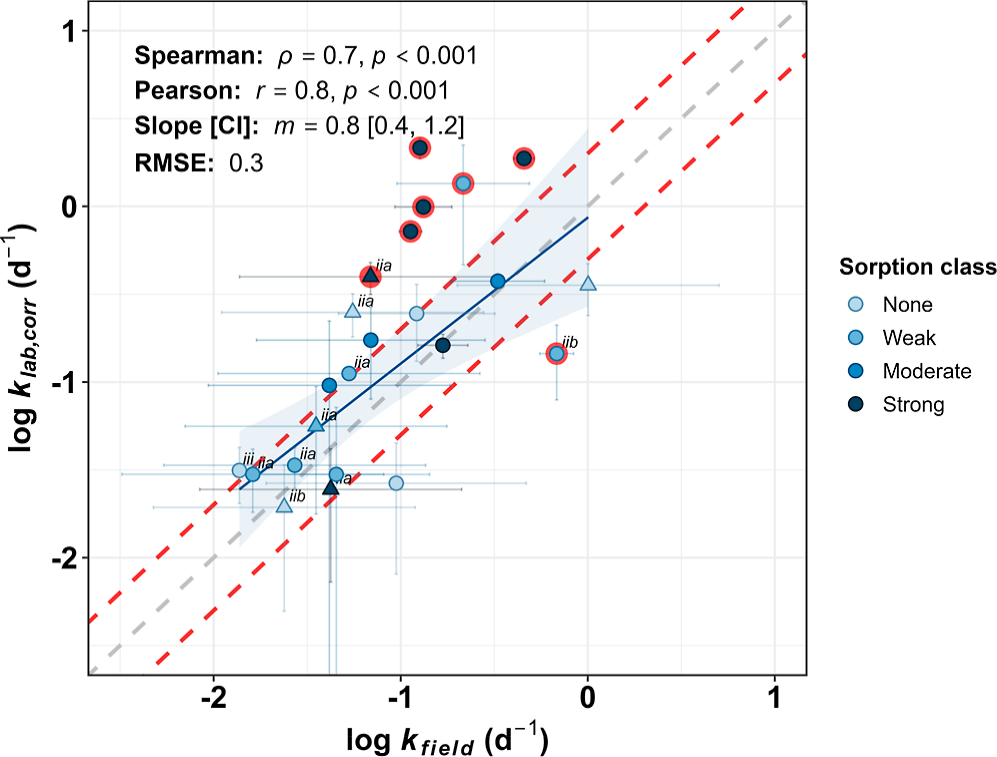
Correlation between log *k*_*field*_ and log *k*_*lab,corr*_. Circular markers represent direct injection-derived *k*_*field*_, while triangular markers represent solid-phase-extraction-derived *k*_*field*_. Vertical error bars represent the confidence intervals of the *k*_*lab,corr*_ calculation and horizontal error bars represent the error-propagated uncertainty in the *k*_*field*_ estimation (95% confidence intervals). Marker colors represent the different sorption classes defined in the *Field Results*. Markers highlighted with red represent outliers (chemicals whose 95% confidence intervals do not intersect the 1:1 line). The solid blue line, dashed gray line, and dashed red lines represent the linear regression between *k*_*lab,corr*_ and *k*_*field*_ for chemicals except those classed as strongly sorbing, the 1:1 relationship, and the ± factor-of-2 bounds around the 1:1 relationship, respectively. Marker text labels represent the data quality of *k*_*field*_, according to the tiers described in the Materials and Methods, where no text label equates to tier (i), i.e., the highest quality. Spearman and Pearson correlation statistics, the slope of the regression and root-mean-square-error (RMSE; log scale) for chemicals except those classed as strongly sorbing are also displayed.

The only previous field-laboratory comparison of relevance is that by Li and McLachlan,^20^ who used a modified OECD 309 test with no addition of either sediment or test chemical (80:20 v/v natural lake water and WWTP effluent; high suspended solids, ∼32 mg L^–1^).^20^ In this hypereutrophic lake, most biodegradation occurred within the water column rather in the sediment. They likewise found close agreement between laboratory and field attenuation (14 of 18 chemicals aligned within a factor of 3).^20^ However, their experimental design could not address the disturbance of the sediment layer and associated redox gradients when sediment is transferred from the field to the laboratory, or situations where surficial sediment plays a dominant role in biodegradation. The present work targets such situations, building on the premise that biodegradation occurs predominantly at the sediment–water interface, and shows that the consequences of disturbing the sediment layer in the modified OECD 309 test were small for nonsorbing to moderately sorbing chemicals. In contrast, strongly sorbing chemicals exhibited systematically faster degradation in the laboratory, which could be due to inhomogeneous sediments combined with nonrepresentative sediment sampling (see above) or marked deviations of the laboratory test from field behavior in such cases (see SI, S7.1 and Figure S5 for further analysis of strongly sorbing chemicals).

There are a number of possible explanations for deviations of the laboratory test from behavior in the field. One chemical, levetiracetam (LTC), fell below the 1:1 line by approximately 5-fold. Such a deviation would be expected if processes causing significant attenuation in the field are absent from- or disrupted during-the laboratory test. Diurnal modulation where attenuation is higher by day due to temperature and/or daylight-linked ecosystem metabolism could occur in the field but not under the constant dark and temperature-controlled conditions of the laboratory experiment. Hyporheic-zone processing is also not represented in the laboratory study: resuspension and oxygenation of the sediment/water slurry removes redox and mass-transfer gradients such that suboxic biofilm/sediment reactions are less likely to occur^32,35,36^ (although anoxic microenvironments may persist within sediment particles^37–40^ ). We note that LTC demonstrated significant temporal trends in signal in the field (tier (ii*b*)), and therefore *k*_*field*_ possesses greater methodological uncertainty.

The remaining outliers were weak bases and fell approximately 5-fold above the 1:1 line (faster *k*_*lab,corr*_) including levamisole (LAS), atenolol (ATE) bisoprolol (BIS), metoprolol (MPL), and nicotinamide (NIC), with the exception of venlafaxine (VEN) which was approximately 12-fold faster in *k*_*lab,corr*_. For LAS, a nonsignificant difference between the upstream and downstream sites (tier (ii*a*)) together with direct-injection measurements below LOD implies that *k*_*field*_ is very uncertain. For NIC, uncertainty in *k*_*lab,corr*_ was high with error bars intersecting the factor-of-two bounds. Uncertainty was low for the remaining outliers. Resuspension in the laboratory experiment could inflate *k*_*lab,corr*_ for chemicals whose field transformation is limited by mass-transport or microbial accessibility. As previously discussed, most strongly sorbing chemicals fell significantly above the 1:1 line, supporting the possibility that differences in mixing and bioavailability between the well-mixed laboratory system and field environment contribute to the observed divergence. In addition, several outliers shared common functional groups (i.e., ATE, BIS, and MPL are all classed as propanolamines^22^), suggesting that degradation pathways for this chemical class may be favored under the laboratory conditions tested. Alternatively, since these outliers were weak bases, the divergence could reflect an overcorrection for the influence pH differences between the laboratory and the field and its impact on the internal concentration in microorganisms;^41–43^ however, this same correction improved agreement for several other chemicals (see SI, S7.2). As such, the overcorrection could be chemical-specific rather than a systematic source of uncertainty. Clearly, there is a range of possible explanations for the seven outliers (in addition to inhomogeneous sediments combined with nonrepresentative sediment sampling).

In summary, the preservation of the rank order of chemical biodegradation (Spearman *ρ* = 0.7, *p* < 0.001) indicates that the modified OECD 309 laboratory test can effectively prioritise the biodegradation of nonsorbing to moderately sorbing chemicals as it would occur in the field. This supports our first hypothesis (H1), that the relative biodegradation rate constants of chemicals is preserved between the laboratory and field experiments, within this chemical domain. The strong Pearson correlation (*r* = 0.8, *p* < 0.001), with the slope of the linear regression close to unity (*m* = 0.8), indicates that the modified OECD 309 laboratory test can approximate environmentally relevant biodegradation kinetics for a range of nonsorbing to moderately sorbing chemicals (RMSE of 0.3, equivalent to a 2-fold difference). This supports our second hypothesis (H2), that biodegradation rate constants in the field can be predicted from the laboratory using a single scaling factor, within this chemical domain. In this case the scaling factor was ∼1.

Several sources of uncertainty, however, constrain the strength of our conclusion that the modified OECD 309 method can reasonably approximate field biodegradation for nonsorbing to moderately sorbing chemicals. First, there was no replication for the field study that permits a direct estimate of precision. Second, the temporal offset of the upstream (WIL) and downstream (OAK) sample collection did not reflect the water travel time in the field study. Our data evaluation assumes approximate steady-state conditions in the streamflow and chemical inputs prior to and during the study period, assumptions that may not have been valid for all chemicals. Third, our comparison was conducted at one site and time. Whether similar alignment of rate constants between the field and laboratory experiments occurs in other aquatic environments and during other seasons remains unknown and will require further testing.

### Regulatory Considerations

An important consideration from a regulatory perspective is the laboratory test’s performance relative to the persistence threshold for chemicals in water. The EU REACH specifies a half-life of 40 days in water, which corresponds to a *k* of approximately 0.017 d^–1^ (log *k* of approximately −1.8 d^–1^). In our data set, several laboratory and field estimates of *k* lay close to this threshold, and agreement for these data points was particularly good (typically within a factor of 2). This indicates that our conclusions regarding H1 (rank-order agreement) and H2 (scaling) were applicable to slowly degrading chemicals near regulatory decision points. However, one caveat arises from the systematic positive bias observed for strongly sorbing chemicals. Although the strongly sorbing chemicals examined in this study were not near regulatory decision limits, laboratory-derived *k* were substantially higher than field-derived *k*, suggesting that the laboratory test may not be precautionary in nature for strongly sorbing chemicals.

Importantly, our laboratory biodegradation rate constants apply to an aquatic system as opposed to a pelagic compartment or a sediment compartment in such a system. Therefore, direct comparison of the laboratory test result with a threshold defined for a pelagic environment is not straightforward. Based on literature and our experience, the half-lives of the studied chemicals in a pelagic test system would be much longer and would likely lie above the regulatory threshold. However, for the assessment of environmental persistence it would seem more relevant that our field test showed that none of the chemicals were persistent in Gowrie Creek (i.e., the aquatic system). Therefore, the mixed sediment/water modified OECD 309 test appears to provide more environmentally relevant estimates of environmental biodegradation than a pelagic test.

## Supplementary Material





## Notes

Summary of attenuation kinetics derived from literature (sheet 1, *Literature Rate Constants*) and biodegradation kinetic parameters of test chemicals quantified in the present study (sheet 2, *Field Rate Constants*; sheet 3, *Laboratory Rate constants*) (XLSX) (XLSX)
Details on the sampling site, materials, methods, and data evaluation procedures, as well as tables and figures containing complementary results (PDF) (PDF)
