# Supplementary material for: Biodegradation Rates of Organic Chemicals in a Subtropical River: From Laboratory to Field
Source: Environ Sci Technol. 2026 Apr 1;60(14):11056–64. doi: 10.1021/acs.est.5c18446 (PMC13085798; doi:10.1021/acs.est.5c18446)
Supplement: Supplementary file 2 [file es5c18446_si_002.pdf]

Supplementary Information

Biodegradation Rates of Organic Chemicals in a Subtropical River: From Laboratory to Field

Lily M. Weir [1], Run Tian [2], Malte Posselt [2], Jochen F. Mueller [1], Michael S. McLachlan [2].*

[1] Queensland Alliance for Environmental Health Sciences (QAEHS), The University of Queensland, 20 Cornwall Street, Woolloongabba, Queensland 4102, Australia

[2] Department of Environmental Science (ACES), Stockholm University, 106 91 Stockholm, Sweden

*Corresponding author: michael.mclachlan@aces.su.se

Number of pages: 30

Number of tables: 6

Number of figures: 9

Table of Contents

S1. Chemical details....4
S2. Site details and hydrology 10
S2.1 Site overview....10
S2.2 Water residence time....12
S2.3 Dilution factor 15
S3. Chemical analysis 16
S4. Quality Assurance and Quality Control (QAQC)....17
S5. Biodegradation kinetics 19
S5.1 Laboratory experiment....19
S5.2 Field experiment 19
S6. Literature-derived k_{field} comparison 20
S7. Laboratory-field comparison 22
S7.1 Impact of strong sorption 22
S7.2 Impact of pH correction 24
S7.3 Impact of k_{lab} data choice....26
S7.4 Impact of photodegradation 28
S7.5 Evaluation of the theoretical framework and implications for laboratory-to-field extrapolation....30
References....31

List of Tables

Table S1: List of test chemicals including abbreviations (Abbr.). S5
Table S2: List of internal standards S9
Table S3: Sampling site details. S11
Table S4: Homogenised surface water and sediment properties at the sites S11
Table S5: Water residence time (τ) estimates between reaches of Gowrie Creek S14
Table S6: Summary of photodegradation behaviour for selected chemicals.........................S26

List of Figures

Figure S1: Timeseries of water quality data S13
Figure S2: Timeseries of discharge data at Cranley (CRA) and Oakey (OAK)....................S16
Figure S3: Comparison of k_{field} derived from direct injection (DI) and solid-phase.............S18
Figure S4: Correlation between rate constants derived from literature S21
Figure S5: Correlation between log k_{field} and log $k_{lab,corr}$, highlighting the behaviour of strongly sorbing chemicals...S23
Figure S6: Correlation between k_{field} and $k_{lab,corr}$, where $k_{lab,corr}$ was not corrected S25
Figure S7: Correlation between k_{field} and $k_{lab,corr}$, where $k_{lab,corr}$ was derived from sediment S27
Figure S8: Correlation between k_{field} and $k_{lab,corr}$, where chemicals suspected to photodegrade are included..S28
Figure S9: Conceptual and empirical evaluation of the influence of sediment-water partitioning on laboratory-to-field biodegradation rate constant extrapolation..S30

S1. Chemical details

The 129 chemicals listed in Table S1 were studied based on criteria outlined in previous works [1,2]. In brief, we targeted chemicals that (1) occur in wastewater-impacted aquatic environments, (2) have low volatility, (3) have low sorption potential (log D_{OW} < 3 at pH 7.4 for 80 % of chemicals), and (5) span a range of expected biodegradability. The chemicals encompassed a range of use categories including pharmaceuticals, personal care products, pesticides, food additives, and industrial chemicals. The chemicals also covered a range of anticipated primary biodegrading functional groups including acetanilides, carboxylic acids, phenylureas, propanolamines, sulfonamides, and thioethers.

A 1.75 µg mL^{-1} working solution of chemicals in Milli-Q water was prepared at the Department of Environmental Sciences (ACES), Stockholm University. A 1 µg mL^{-1} internal standard solution in methanol (containing isotope-labelled standards, see Table S2) was also prepared. All standards were frozen at -20 °C prior to use. Other reagents including LC/MS-grade methanol and formic acid were purchased from VWR and Sigma-Aldrich, respectively (Stockholm, Sweden). Sodium azide and sodium pyrophosphate were purchased from Sigma-Aldrich (Brisbane, Australia). Milli-Q water was sourced from a Milli-Q Water Purification System (Merck Millipore, Brisbane, Australia and Stockholm, Sweden).

Table S1: List of test chemicals including their abbreviations (Abbr.). Modified from Tian *et al.* [1]. Dissociation constants sourced from PubChem, DrugBank, and ChemBK. pH-dependent octanol-water distribution coefficient (log D_{OW}) sourced from ChemSpider.

	**Chemicals**	**Abbr.**	**CAS Number**	**log D_{OW} (pH 7.4)**	**p*K*a**
1	1-Stearoyl-rac-glycerol	1ST	123-94-4	2.6	-
2	2,6-Di-tert-butyl-4-methylphenol	2DI	128-37-0	5.07	11.6[a]
3	3-((2-Ethylhexyl)oxy)propane-1,2-diol	3ET	70445-33-9	2.4	13.64[a]
4	3-Methyl-4-(2,6,6-trimethyl-2-cyclohexen-1-yl)-3-buten-2-one	3ME	127-51-5	4.22	-
5	4-(4-Nitrobenzyl)-pyridine	4NP	1083-48-3	2.6	5.51[b]
6	4-Chloro-3,5-dimethylphenol	4CD	88-04-0	2.83	9.7[b]
7	5-Methylbenzotriazole	5MB	136-85-6	1.69	8.74[b]
8	Abacavir	ABA	136470-78-5	1.32	5.8[b]
9	Acesulfame	ASF	33665-90-6	-2.77	2.0[a]
10	Acetamiprid	ATP	135410-20-7	1.06	0.7[a]
11	Alachlor	ALA	15972-60-8	2.99	1.20[a]
12	Amisulpride	AMI	71675-85-9	-0.43	9.37 [b]
13	Anastrazole	ANA	120511-73-1	2.68	1.4 [b]
14	Atazanavir	ATA	198904-31-3	4.61	4.42 [b]
15	Atenolol	ATE	29122-68-7	-1.85	9.58 [b]
16	Atrazine	ATR	1912-24-9	2.66	4.2 [b]
17	Azoxystrobin	AZO	131860-33-8	3.54	1.94[b]
18	Benzotriazole	BTZ	95-14-7	1.5	8.37[b]
19	Benzyl alcohol	BEA	100-51-6	4.85	15.4[a]
20	Benzyl salicylate	BES	118-58-1	4.01	8.[11b]
21	Bezafibrate	BEZ	41859-67-0	-0.11	3.83[a]
22	Bisoprolol	BIS	66722-44-9	0.12	9.67[b]
23	Bromoxynil	BRO	1689-84-5	3.77	3.86[a]
24	C12 Isethionate	CIS	3/01/7381	-	-
25	Caffeine	CAF	58-08-2	0.28	-1.2[b]
26	Candesartan	CAN	139481-59-7	0.04	2.45[b]
27	Carbamazepine	CBZ	298-46-4	2.28	13.9[a]
28	Carbendazim	CAR	10605-21-7	1.51	4.29[b]
29	Chlorothiazide	CTZ	58-94-6	-0.21	4.29[a]
30	Chlorthalidone	CTD	77-36-1	0.41	6.85[a]
31	Chlortoluron	CTU	15545-48-9	2.48	14.43[a]
32	Cilastatin	CIL	82009-34-5	-1.9	9.14[b]
33	Ciprofloxacin	CIP	85721-33-1	-2.23	8.77[b]
34	Citalopram	CIT	59729-33-8	1.27	9.78[b]
35	Climbazole	CLI	38083-17-9	3.32	6.49[b]

36	Clofibric acid	CLA	882-09-7	-0.88	-
37	Cocoamidopropyl betaine	COC	61789-40-0	0.93	-
38	Cyclamate	CYC	100-88-9	-3.51	1.7[b]
39	Decylamine	DEC	2016-57-1	1.07	10.64[b]
40	Dibenzepine	DIB	4498-32-2	1.24	8.23[b]
41	Dicamba	DAB	1918-00-9	1.24	1.97[b]
42	Diclofenac	DIC	15307-86-5	1.37	1.97[a]
43	Diflufenican	DIF	83164-33-4	4.09	9.03[b]
44	Dimethenamid	DIM	87674-68-8	2.45	1.16[b]
45	Dioctyl Sulfocinate Sodium Salt	DIO	10041-19-7	1.01	-0.75[a]
46	Diuron	DIU	330-54-1	0.13	-
47	Dodecyl sulfate sodium salt	DSS	151-21-3	1.6	-1.5[a]
48	Dodecylamine	DOD	124-22-1	1.86	10.63[b]
49	Dodecyltrimethylammonium chloride	DOC	112-00-5	-	-
50	Ethofumesate	EFS	26225-79-6	2.14	-
51	Ethylene glycol butyl ether	EGB	111-76-2	0.77	14.42[a]
52	Ethylhexyl methoxycinnamate	EMC	5466-77-3	5.28	-4.8[b]
53	Ethylparaben	EPB	120-47-8	2.48	8.34[a]
54	Fenhexamid	FHX	126833-17-8	4.17	7.3[a]
55	Fenofibrate	FFE	49562-28-9	5.01	-4.9[b]
56	Fipronil	FIP	120068-37-3	3.71	-5.86[b]
57	Flecainide	FLE	54143-55-4	1.01	9.3[b]
58	Fluconazole	FCZ	86386-73-4	0.7	1.76[b]
59	Fludioxonil	FDO	131341-86-1	2.57	14.10[a]
60	Flufenacet	FFA	142459-58-3	3.01	0.31[a]
61	Fluoxetine	FXT	54910-89-3	1.75	9.8[b]
62	Furosemide	FUR	54-31-9	-0.78	3.65[a]
63	Gabapentin	GAB	60142-96-3	-1.4	3.7[a]
64	Galaxolide	GAL	1222-05-5	5.93	-
65	Gemfibrozil	GEM	25812-30-0	1.58	4.5[a]
66	Hexylene glycol	HGL	107-41-5	0.23	15.10[a]
67	Homosalate	HOM	118-56-9	5.23	9.72[a]
68	Hydrochlorothiazide	HCZ	58-93-5	-0.01	7.9[a]
69	Hydroxy bupropion	HBP	357399-43-0	1.93	-
70	Imidacloprid	IMI	138261-41-3	-0.29	1.56[b]
71	Iodopropynylbutylcarbamate	IBC	55406-53-6	3.2	14.4[a]
72	Iprovalicarb	IPO	140923-17-7	3.29	11.41[a]
73	Irbesartan	IRE	138402-11-6	1.24	5.85[a]
74	Isoproturon	ISO	34123-59-6	2.45	15.06[a]
75	Ketoprofen	KET	22071-15-4	0.06	3.98[a]

76	Lamotrigine	LAM	84057-84-1	1.68	5.7[b]
77	Levamisole	LAS	14769-73-4	0.25	6.98[b]
78	Levetiracetam	LTC	102767-28-2	-0.74	-1.6[b]
79	Lidocaine	LID	137-58-6	1.26	7.95[b]
80	Linezolid	LIN	165800-03-3	0.82	1.8[b]
81	Losartan	LOS	114798-26-4	1.29	5.5[a]
82	Loxynil	LOX	1689-83-4	1.27	3.96[a]
83	MCPA	MCPA	94-74-6	-1.09	3.13[a]
84	Mecoprop	MEC	7085-19-0	-0.65	3.21[a]
85	Mefenamic acid	MEF	61-68-7	2.04	4.2[a]
86	Metformin	MET	657-24-9	-3.36	12.4[b]
87	Methotrexate	MEX	59-05-2	-5.22	4.7[a]
88	Methyl 4-hydroxybenzoate	M4H	99-76-3	2.09	8.5[a]
89	Metolachlor	MLC	51218-45-2	3.22	1.45[a]
90	Metoprolol	MPL	51384-51-1	-0.25	9.56[b]
91	Metoxuron	MXR	19937-59-8	1.87	13.83[a]
92	N-(3-dimethylaminopropyl)-octadecanamide	NDO	7/02/7651	6.12	16.29[a]
93	N,N-bis(2-hydroxyethyl)dodecanamide	NHD	120-40-1	3.95	14.13[a]
94	N,N-Bis(2-hydroxyethyl)tetradecanamide	NHT	7545-23-5	4.87	-
95	Naphthalene-2-carbonitrile	N2C	613-46-7	3.22	-
96	Naproxen	NAP	22204-53-1	0.45	4.15[a]
97	Neostigmine	NEO	59-99-4	-2.77	-
98	Nicotinamide	NIC	98-92-0	-0.37	3.35[b]
99	Novobiocin	NOV	303-81-1	-0.02	4.3[a]
100	Oxazepam	OXA	604-75-1	1.5	1.55[b]
101	Oxprenolol	OXP	6452-71-7	0.19	9.57[b]
102	Paracetamol	PAR	103-90-2	0.4	9.38[b]
103	Pargyline	PGL	555-57-7	2.31	8.05[b]
104	Picoxystrobin	PIC	117428-22-5	3.84	-1.09[a]
105	Propachlor	PPC	1918-16-7	2.28	0.30[a]
106	Propranolol	PPN	525-66-6	1.15	9.53[b]
107	Propyl 4-hydroxybenzoate	P4H	94-13-3	2.81	8.5[a]
108	p-Toluenesulfonic acid	PTA	104-15-4	-3.36	-1.34[a]
109	Ranitidine	RAN	66357-35-5	-0.63	7.8[b]
110	Rufinamide	RUF	106308-44-5	0.42	12.64[a]
111	Sodium decyl sulfate	SDS	142-87-0	-	-
112	Sorbitan monolaurate	SML	1338-39-2	4.02	-
113	Sotalol	SOT	3930-20-9	-1.63	9.76[a]

114	Sulfadimethoxine	SMX	122-11-2	-0.49	6.91[a]
115	Sulfamethazine	SMT	57-68-1	0.79	7.59[a]
116	Sulfamethoxazole	SMZ	723-46-6	-0.56	6.16[a]
117	Sulfamethoxypyridazine	SMP	80-35-3	-0.29	6.84[a]
118	Sulfathiazole	STZ	72-14-0	0.03	7.2[a]
119	Tamsulosin	TAM	106133-20-4	0.77	9.28[b]
120	Tartrazine	TAR	1934-21-0	-10.17	-
121	Terbutryn	TBR	886-50-0	1.38	4.30[b]
122	Tetradecylamine	TAL	2016-42-4	3.05	10.62[a]
123	Tramadol	TRA	27203-92-5	0.52	9.41[b]
124	Triethylcitrate	TRC	77-93-0	1.09	11.82[a]
125	Trimethoprim	TMP	738-70-5	-1.15	7.12[b]
126	Trinexapac-ethyl	TAE	95266-40-3	-1.76	4.7[a]
127	Valsartan	VAL	137862-53-4	-0.89	3.6[a]
128	Venlafaxine	VEN	93413-69-5	1.43	9.5[b]
129	Zolpidem	ZOL	82626-48-0	3.06	5.65[b]

[a]acid p*K*a, [b]base p*K*a.

Table S2: List of internal standards.

	**Internal Standards**
1	2-hydroxy-Ibuprofen-d6
2	acesulfame-d4
3	acetaminophen-d4
4	atenolol-d7
5	atorvastatin-d5
6	bezafibrate-d4
7	caffeine-d9
8	climbazole-d4
9	carbamazepine-d8
10	clofibric acid-d4
11	conitine-(methyl-d3)
12	fluconazole-d4
13	gabapentin-d6
14	gemfibrozil-d6
15	glimepiride-d5
16	irbesartan-d6
17	ketoprofen-13C, d3
18	MCPA-d6
19	mecoprop-d3
20	metformin-d6
21	metoprolol acid-d5
22	metoprolol-d7
23	naproxen-d3
24	oxazepam-d5
25	pravastatin-d3
26	propranolol-d7
27	sulfamethoxazole-d4
28	tramadol-d6
29	triethyl-d15-phosphate
30	valsartan-d3
31	venlafaxine-d6
32	zolpidem-d6

S2. Site details and hydrology

S2.1 Site overview

Gowrie Creek drains the city of Toowoomba on the Great Dividing Range (QLD, Australia) and flows westward/inland via Oakey Creek to the Condamine River in the Murray-Darling Basin. The catchment is predominantly urban within Toowoomba, with mixed peri-urban and agricultural land uses downstream [3,4]. The principal point source is the Wetalla Wastewater Treatment Plant (WWTP, total daily design capacity of 36 ML day^{-1}) discharging to Gowrie Creek [5]; treated effluent contributes to the Upper Condamine system [6]. The climate is sub-tropical upland: mean annual rainfall ~700 mm, concentrated between November and March, and with temperate conditions at higher elevations in the catchment (Bureau of Meteorology, Toowoomba airport records [7]).

Gowrie Creek was selected because it has a single dominant point source (WWTP) which discharges to a long downstream reach with no major additional inputs. This configuration allows a simplified conceptual model of the experiment and estimation of in-stream attenuation from paired upstream-downstream observations. Hydraulic stability was sought secondarily to reduce mass-balance uncertainty.

We surveyed multiple potential sampling locations along Gowrie Creek to assess suitability for the present study. From this assessment, two sites downstream of the WWTP discharge were selected for the field study and equipped with autosamplers (WIL and OAK). In parallel, three sites were designated for the laboratory experiment, where water and sediment were collected for the modified OECD incubations (WIL, JUN and OAK). In addition, a site upstream of the WWTP discharge (CRA) was monitored for hydrological purposes. The WIL-OAK study reach is approximately 35 km approximate middle thread distance (AMTD) long. Both studies were conducted in May 2023. All sites are depicted in Figure 1 (main text), and their details are listed in Table S3 and Table S4.

Table S3: Sampling site details.

Waterway	Site Name	Site Name Abbr.	AMTD (km)*	Latitude	Longitude	Site Equipment		Used in laboratory experiment?
						Autosampler	YSI sonde	
Gowrie Creek	Cranley Gauge	CRA	-2.5	-27.51711	151.94117		x	
	Willims Road	WIL	0.5	-27.50173	151.92672	x	x	x
	Gowrie Junction	JUN	7	-27.49719	151.88900		x	x
	Oakey Gauge	OAK	35	-27.47066	151.74073	x	x	x

* relative to the WWTP discharge location.

Table S4: Homogenised surface water and sediment properties at the sites (laboratory experiment).

Waterway	Sites	Period	Surface Water						Sediment				
			Temperature (^{0} C)	Specific Conductivity (µS cm^{-1})	pH	Dissolved Oxygen (%)	Turbidity (NTU)	Chlorophyll-a (µg/L)	Particle Size Distribution (%)				Organic Carbon (%)
									Clay	Silt	Fine Sand	Coarse Sand	
Gowrie Creek	WIL, JUN, OAK	Field *	19.4	700	8.5	101	6	6	44.1	0.62	28.5	12.2	0.62
		Lab ^	19.6	800	8.0	98	-	5	-	-	-	-	-

* average of measurements across sites.

^ average of all measurements over experiment duration.

S2.2 Water residence time

Water residence time (τ) was estimated by tracking the time-lagged diurnal signals in specific conductivity (SpCond) and water depth generated by the WWTP discharge, as described in the main text. Briefly, semi-continuous measurements were obtained using YSI 6-series sondes (Xylem Water Solutions, Brisbane) deployed at three locations along Gowrie Creek (WIL, JUN, and OAK). These signals were used to constrain advection time within a one-dimensional reactive transport model (see main text for conceptual description and assumptions).

Supporting timeseries of SpCond, water depth, dissolved oxygen, and water temperature are shown in Figure S1. Clear diurnal SpCond signals were observed between WIL and JUN, allowing estimation of travel time using nonparametric deconvolution of the SpCond timeseries. At the downstream site (OAK), attenuation of the SpCond signal prevented reliable model fitting. In this reach, travel time was therefore inferred from the depth signal, which remained coherent over the longer transect. Consistent agreement between SpCond- and depth-derived estimates in the upstream reach supported the use of the depth signal where SpCond data were insufficient. However, since depth fluctuations represent propagation of a hydraulic pressure wave rather than solute transport, travel times derived for the downstream reach may exceed true advective residence time. Accordingly, this estimate was interpreted as a lower bound on τ.

For τ used in the main analysis, SpCond and depth data between the 1st and 12th of May 2023 were fitted (water samples for chemical analysis were collected on the 12th-14th of May 2023. A minimum of six days of data were required for stable deconvolution (although this was dependant on data quality). The τ between WIL and OAK was estimated at 47 ± 3 hours, with uncertainty propagated from temporal variability. To assess temporal variability and method robustness, we repeated the τ analysis post hoc. Sondes were redeployed between September and November 2024. The resulting τ estimates and sensitivity to hydraulic changes are summarised in Table S5.

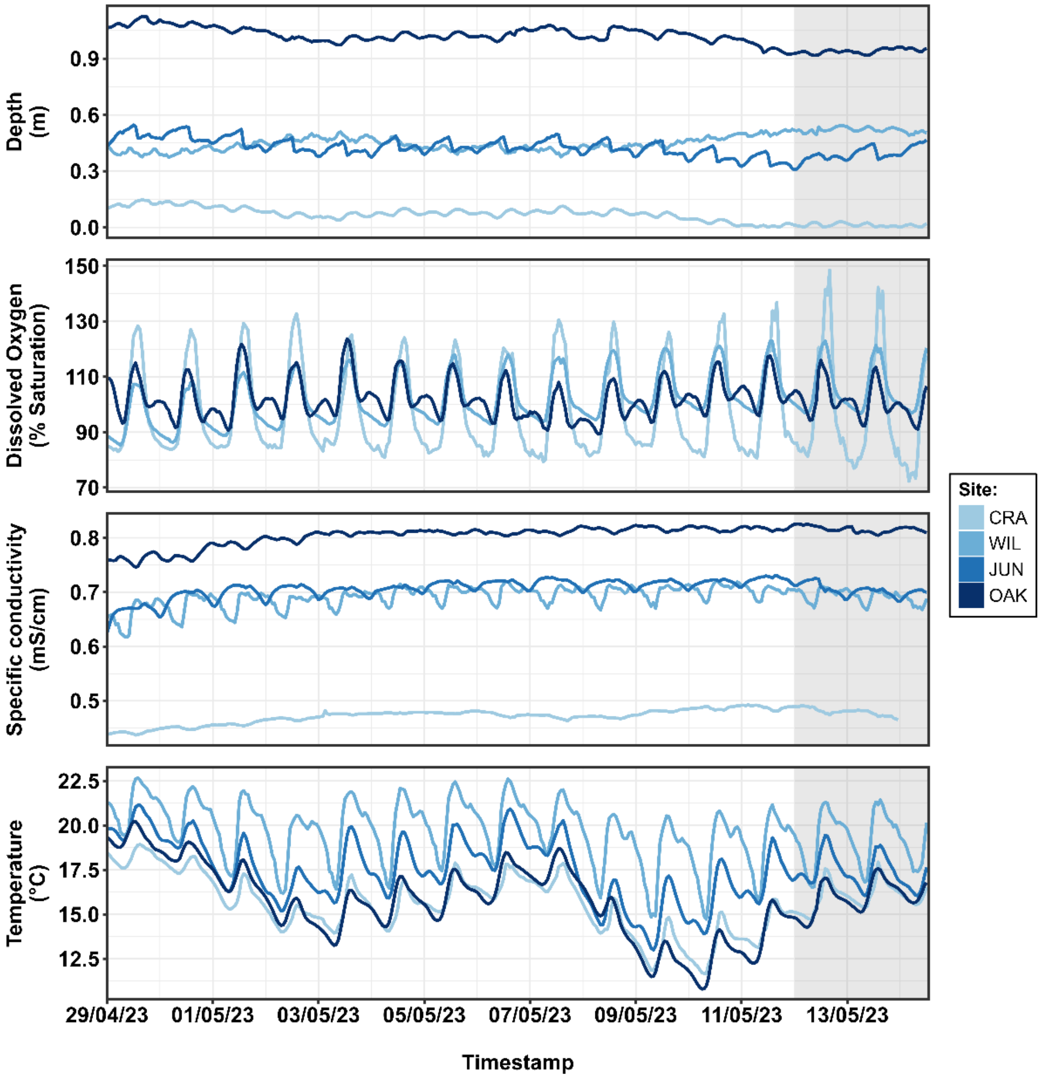

Figure S1: Timeseries of water quality data (depth, dissolved oxygen, specific conductivity, and temperature) from continuously-logging water quality sondes (Xylem Water Solutions, Brisbane) deployed at sites along Gowrie Creek. Grey shaded boxes represent the period of autosampler deployment.

Table S5. Water residence time (τ) estimates between reaches of Gowrie Creek.

Reach	Method	Data	Period	**τ (h)**
CRA – WWTP	1D plug-flow	Gauge Q	01/05/23-12/05/23	12
			20/09/24-06/10/24	7
			13/10/24-5/11/24	9
WIL – JUN	1D reactive transport model	SpCond	01/05/23-12/05/23	6
		Depth		6
		SpCond	20/09/24-06/10/24	7
		Depth		11
		SpCond	13/10/24-5/11/24	7
		Depth		7
JUN – OAK	1D reactive transport model	Depth	01/05/23-12/05/23	41
		Depth	20/09/24-06/10/24	37
		Depth	13/10/24-5/11/24	42

S2.3 Dilution factor

To quantify the dilution factor (*df*) for the WWTP-derived chemicals during the autosampler deployment window, we applied a water residence time-aligned mass balance of discharges. Discharge data were obtained from the Cranley (CRA) and Oakey (OAK) gauges (Queensland Government Department of Local Government, Water and Volunteers) [8], and the WWTP. Pre-screening of gauge and rainfall data over the two weeks preceding and including the study period indicated baseflow conditions (i.e., no rainfall runoff contribution, see Figure S2).

For each downstream observation at time *t*, we aligned contributing hydrographs by the water residence time (τ) according to Eqn S1. Given that the discharge upstream of the WWTP (i.e., at CRA gauge) was highly stable prior and during the period of interest (variation < 10%), we did not attempt additional temporal alignment for this segment beyond τ measured from WIL to OAK as any further refinement would rely on assumptions that are not well constrained and would not materially reduce the overall uncertainty.

$$df(t) = \frac{Q_{CRA}(t - \tau_{down}) + Q_{WWTP}(t - \tau_{down})}{Q_{OAK}(t)} \qquad \text{(Eqn S1)}$$

where Q_{CRA}, Q_{WWTP}, and Q_{OAK} are discharges (ML d^{-1}), and τ_{down} represents the water residence time from WIL to OAK (~47 hours).

WWTP discharge records showed an average discharge of 25 ML d^{-1} (± 1.5) over this period, while the upstream baseflow contribution averaged 10 ML d^{-1} (± 0.7); thus, wastewater comprised ~60 % of dry-weather flow. At the downstream gauge co-located with OAK, measured discharge exceeded the sum of upstream discharges by ~10 % (i.e., *df* = 1.1 ± 0.02), potentially due to diffuse groundwater inputs from the Main Range Volcanic aquifer [9,10]. Literature for this system under comparable conditions reported wastewater fractions > 80 % persisting tens of kilometres downstream and conservative WWTP-tracer persistence (i.e., gadolinium) > 50 km [6].

Persistent organic micropollutants, including but not limited to PFAS, have been used previously as tracers [11-13]. As a cross-validation, we compared discharge-derived *df* to conservative PFAS tracer ratios. Assuming negligible in-reach sources and sinks of persistent PFAS *i*, dilution can be estimated using the average of the drift-corrected peak areas ($\bar{A}$) at WIL and OAK according to Eqn S2.

$$\widehat{df}_{PFAS,i} = \frac{\bar{A}_{i,WIL}}{\bar{A}_{i,OAK}} \qquad \text{(Eqn S2)}$$

Samples from the same sites and period were analysed following SPE using a targeted PFAS method [14,15], yielding an average *df* of 1.1 ± 0.5 across five PFAS (PFBS, PFDA, PFHxS, PFOS and PFNA). This was compared with the *df* derived for the SPE samples corresponding with the chemical data used in the present study analysed on the Orbitrap in negative mode (noting that this method did not include PFAS-specific standards), which gave a similar average *df* estimate of 1.2 ± 0.2 across the same five PFAS. This agreed with discharge-derived *df* given the uncertainty in

the tracer-based estimate and supports the use of the discharge-derived *df* for correcting the downstream peak areas of chemicals.

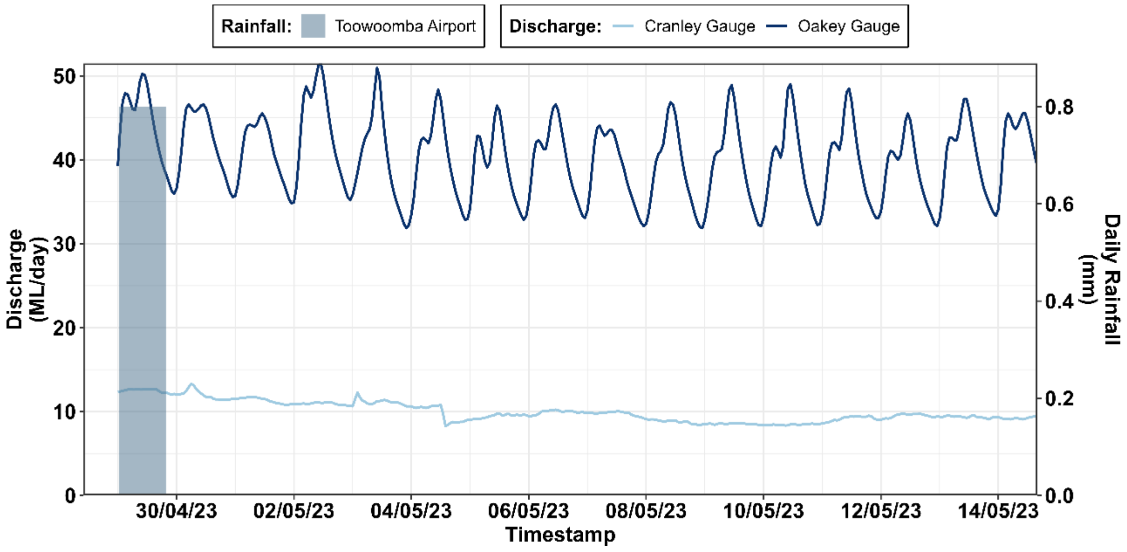

Figure S2: Timeseries of discharge data at Cranley (CRA) and Oakey (OAK) gauges on the primary y-axis (light and dark blue lines), and rainfall data on the secondary y-axis (grey bars). Gauging station data summarises hourly discharge measurements, while rainfall data represents the daily totals. Rainfall data sourced from the Bureau of Meteorology (Australian Government). Discharge data sourced from the Department of Local Government, Water and Volunteers (Queensland Government).

S3. Chemical analysis

Chemical analysis for both field and laboratory experiments was conducted using ultrahigh-performance liquid chromatography coupled to a Q Exactive HF Hybrid Quadrupole-Orbitrap mass spectrometer (UHPLC-Orbitrap-MS/MS) with electrospray ionization (ESI) as described by [16]. Both field and laboratory experiment samples were spiked with a mixture of 32 mass-labelled internal standards (Table S2) prior to their direct injection. In addition, 250 mL of field samples were also spiked with the same mixture of mass-labelled internal standards, concentrated through solid-phase-extraction (SPE; Oasis HLB) following methods by Li *et al.* [17] and Li *et al.* [18], and then injected. Data from both experiments were processed in Compound Discoverer 3.3.

S4. Quality Assurance and Quality Control (QAQC)

Mass calibration of the Orbitrap was performed prior to each sequence. Sample order during the sequences was randomised to eliminate systematic errors associated with the instrument. Matrix quality control (QC) samples were created using a mixture of filtered river water collected at three sites along Gowrie Creek, spiked with 1 ug L^{-1} of the native chemical mix and 10 ug L^{-1} of internal standards. To assess instrument drift, a QC sample was measured at the beginning of each sample batch, after every 6 samples and at the end of each sample batch. An R package, *batchCorr* (version 0.2.5) [19], was used to correct the sample batch data for the drift observed in the QCs. A 15-point matrix-matched calibration curve was created with concentrations spanning 1 ng L^{-1} to 10 ug L^{-1}. The limit of detection for peak area was set at the lowest detected calibration standard, while the limit of quantification was set at the lowest detected calibration standard within the linear range of the calibration curve. The following QAQC metrics are specific to the field study/samples. Details regarding the laboratory study/samples have been published elsewhere [20].

After drift-correction, the relative standard deviation (RSD) in the QC samples was < 30 % for 90 % of native chemicals (median RSD of 26 %) and 100 % of internal standards (median RSD of 8 %). Furthermore, for directly injected samples, the RSD was < 30 % for 86 % of internal standards (median RSD of 9 %), and for SPE-extracted samples, the RSD was < 40 % for 73 % of internal standards (median RSD of 32 %).

We compared DI and SPE estimates of k (i.e., $k_{field,DI}$ and $k_{field,SPE}$, respectively) per chemical using the 95 % confidence interval (CI) of the log-difference in k (Figure S3). For the majority of chemicals, the 95 % CI of k encompassed zero (i.e., DI and SPE were not distinguishable at 95% CI). Eight of the 34 comparable chemicals were flagged as outliers (i.e., the 95 % CI excluded zero), suggesting chemical-specific extraction effects. Only one of these outliers, bisoprolol (BIS), was relevant to the final field dataset. These results indicate that the typical deviation between DI and SPE-derived k was relatively small and not statistically significant. Accordingly, DI and SPE were used interchangeably.

We explored the impact of internal-standard correction for chemicals with matching internal standards. This correction reduced the RSD in the QC samples by ~20 %. However, it had limited impact on the variation across samples from the same site (median change of 1 %), which suggested that temporal variability exceeded variability introduced by matrix interferences. The influence of internal standard correction on k_{field} had a bias (there was a median decrease of k_{field} of 30 %), which could conceivably result in increased between-chemical variability when internal standard corrected and uncorrected chemicals are compared in Figure 2 (matching internal standards were only available for 8 of 24 chemicals). However, this effect was small; the Spearman and Pearson coefficients and the slope were unchanged (0.8, 0.8 and 0.9, respectively) when the uncorrected k_{field} data were used for all chemicals.

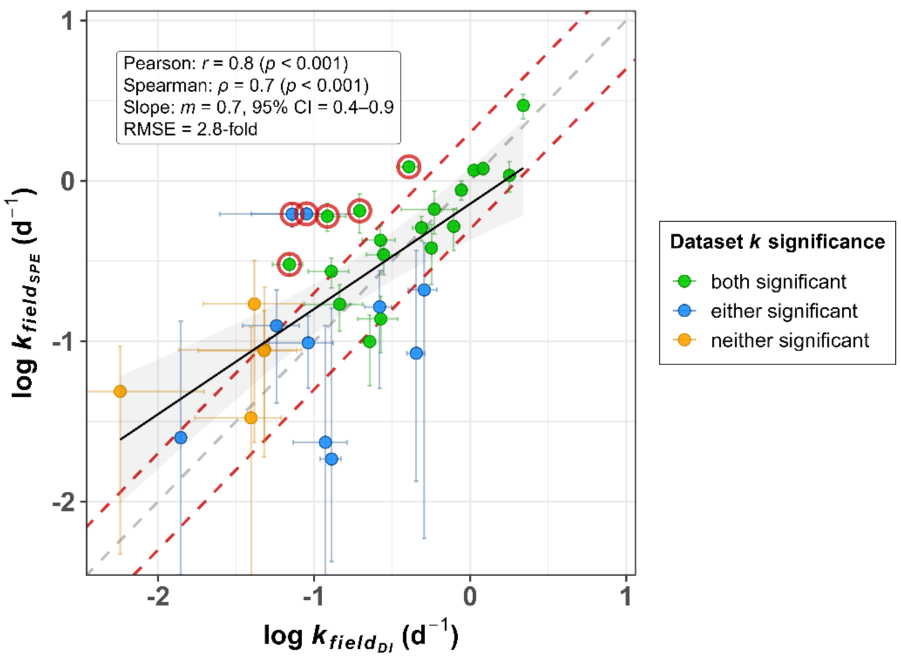

Figure S3: Comparison of k_{field} derived from direct injection (DI) and solid-phase-extracted (SPE) samples (i.e., $k_{field,DI}$ and $k_{field,SPE}$, respectively). Error bars represent the error-propagated uncertainty in the k_{field} estimation (95 % confidence intervals). The solid black line, dashed grey line, and dashed red lines, represent the linear regression between $k_{field,DI}$ and $k_{field,SPE}$, the 1:1 relationship, and the ± factor-of-2 bounds around the 1:1 relationship, respectively. Marker colours represent whether k_{field} was significant for either, both, or neither datasets (t-tests on the peak areas between sites used to test the significance of k_{field}, p < 0.05). Spearman and Pearson correlation statistics, the slope of the regression and root-mean-square-error (RMSE; reported on a linear scale) are also displayed.

S5. Biodegradation kinetics

S5.1 Laboratory experiment

Rate constants were calculated via methods developed by Tian *et al.* [1]. Drift-corrected peak areas for each chemical across all flasks were normalised to the peak area at t_0 then natural-log transformed. From here, two datasets were evaluated. The first used the data direct from the test flasks, while the second dataset used the sterilised sediment control flask to correct the test flasks for potential abiotic attenuation. For the latter, the average natural logarithm of the normalised peak area in the sterilised sediment control flasks was subtracted from that in each test flask at each timepoint. The resultant datasets were run through a Python algorithm (*chowclassifier*)[6] which estimated biodegradation rate constants from all three test flasks/replicates simultaneously and included breakpoint identification in the case of biphasic kinetics. When biphasic kinetics occurred, the first biodegradation rate constant was used. Biodegradation rate constants (k_{lab}) were deemed "valid" if they were significantly different from zero (t-test on the linear regression slope coefficients, $p < 0.05$). Of the 129 test chemicals, 104 were detected. For k_{lab} derived from the test flask data, 63 rate constants were valid. For k_{lab} derived from sterlised sediment control flask corrected data, 52 rate constants were valid.

S5.2 Field experiment

Uncertainty in the estimation of k_{field} was quantified through error propagation of log-transformed peak area variability at the upstream (WIL) and downstream (OAK) sites. Because the upstream and downstream peak areas were not taken in perfectly matched pairs, we treated them as independent samples. Site-specific variances of the natural-log peak areas, $\sigma^2_{ln\bar{A}WIL}$ and $\sigma^2_{ln\bar{A}OAK}$, were combined in quadrature to obtain the standard error (SE) of the log-difference and then propagated to k_{field} via division by the average linear velocity of the chemical (U, m h^{-1}) over the distance between sites (d, m), as per Eqn S3. Here, n is the number of replicates for each site.

$$SE(k) = \frac{U}{d}\sqrt{\frac{\sigma^2{}_{ln\bar{A}_{WIL}}}{n_{WIL}} + \frac{\sigma^2{}_{ln\bar{A}_{OAK}}}{n_{OAK}}} \qquad \text{(Eqn S3)}$$

This likely overestimates the random error of k_{field} since the variance of A_{WIL} and A_{OAK} are not calculated from true replicate measurements of chemicals in the same parcel of water; they provide a measure of the temporal variability instead. We formed two-sided 95 % confidence intervals (CI) using a normal (z-based) approximation. To assess the potential impact of temporal drift that could bias the rate constant estimates, we tested for monotonic trends in the site-wise log-transformed signals using Kendall's Tau (τ). Compounds showing significant temporal trends ($p < 0.05$) were flagged for caution in interpretation.

S6. Literature-derived k_{field} comparison

To contextualise the field results, log-transformed rate constants from this study were compared with the average log-transformed rate constants from other published field studies after correction for key environmental differences. The literature data include studies conducted under a wide range of environmental conditions with varying methods and they thus provide a general indication of the degradation rate constants in aquatic environments (primarily freshwater). Literature data were included only when first-order rate constants were reported directly or could be reconstructed from half-lives or attenuation percentages, together with pH, water residence times, and other required metadata. Datasets lacking this minimum information were excluded. All literature rate constants were adjusted to the pH of the present study. For one study in which rate constants were normalised to total organic carbon (Seller *et al.* [21]), the TOC normalisation was removed prior to comparison.

Several studies reported multiple sampling locations, hydrological conditions, or sampling periods. In these cases, all eligible rate constants for a given chemical were compiled and the literature comparator, $k_{literature}$, was taken as the average across the available conditions, with the corresponding standard deviation defining the uncertainty bounds in Figure S4. Chemicals for which only a single $k_{literature}$ estimate was available were retained and are flagged with an asterisk in Figure S4.

Comparison of log $k_{literature}$ and log k_{field} revealed substantial variability among chemicals (Figure S4). Of the 20 chemicals evaluated, five showed agreement within a factor of two and twelve within a factor of ten. Rate constants from literature were, on average, higher than those observed in the present field study: thirteen chemicals exhibited $k_{literature}$ more than two-fold faster than k_{field}. Only two chemicals (5MB and LOS) showed markedly faster degradation in the present field study, noting that the comparison for LOS was based on a single literature datapoint. The median difference between $k_{literature}$ and k_{field} corresponded to approximately a six-fold discrepancy. Consistent with this variability, statistical measures of agreement were weak (Pearson $r = 0.3$; Spearman $\rho = 0.3$; both not significant), with large uncertainty in the regression slope and substantial dispersion (RMSE = 1.4, equivalent to a factor of 23). Given the spatiotemporal variability reported in biodegradation rate constants previously [2,20,22], strong agreement was not anticipated, and these results thus highlight the challenge of extrapolating biodegradation rate constants in space and time.

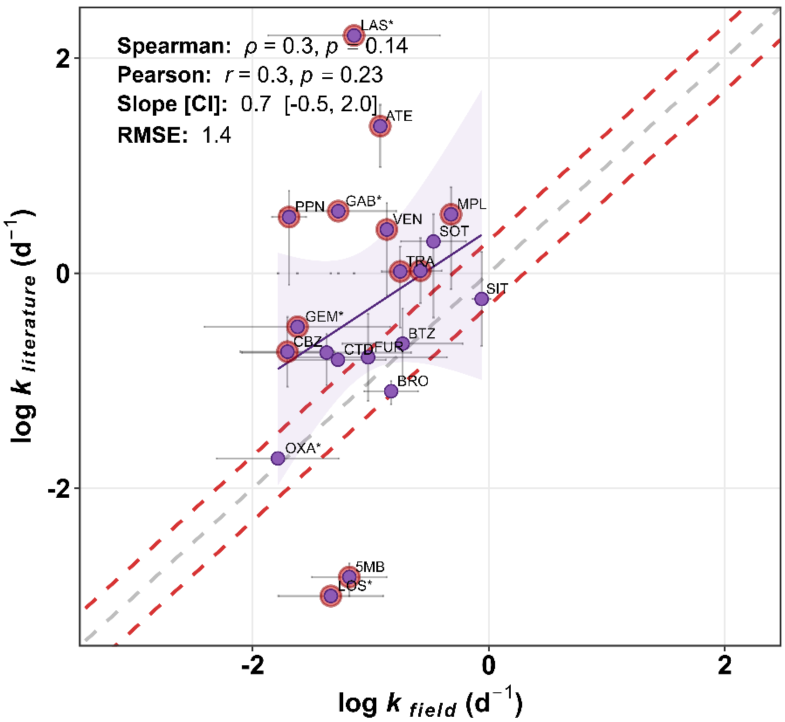

Figure S4: Correlation between average rate constants derived from literature (log $k_{literature}$, pH-corrected) and this study's log k_{field}. Vertical error bars represent the standard deviation in log $k_{literature}$ across studies and horizontal error bars represent the error-propagated uncertainty in the log k_{field} estimation. Markers highlighted with red represent outliers (defined as chemicals whose 95 % uncertainty intervals do not intersect the 1:1 line). The dashed grey line and dashed red lines represent the 1:1 relationship, and the ± factor-of-2 bounds around the 1:1 relationship. Marker text labels denote the abbreviated chemical name (see Table S1). Marker text labels with "*" indicate chemicals for which only one rate constant was available from the literature. Spearman and Pearson correlation statistics, the slope of the regression and root-mean-square-error (RMSE) are also displayed for each scenario.

S7. Laboratory-field comparison

S7.1 Impact of strong sorption

Figure S5 shows the laboratory-field comparison restricted to strongly sorbing chemicals, with the regression for non-sorbing to moderately-sorbing chemicals displayed in purple for reference. As a group, the strongly sorbing chemicals were clearly offset from the 1:1 relationship, with $k_{lab,corr}$ averaging approximately five-fold greater than k_{field}. Variability within this subset was high (RMSE = 0.6, equivalent to a factor of 4), and correlation metrics were non-significant, indicating heterogeneous responses among individual chemicals. Two chemicals (CAF and TRA) fell within a factor-of-two agreement, however, interpretation for one of these (CAF) is limited by k_{field} uncertainty (SPE-derived analytical method and temporal trends in the field data). One explanation for the offset behaviour of these strongly sorbing chemicals is that sediment disturbance or enhanced mixing in the laboratory experiment may preferentially accelerate degradation of such chemicals relative to field conditions. However, since chemicals within this cluster are weak bases, and three of these are structurally related (propanolamines including atenolol [ATE], bisoprolol [BIS], and metoprolol [MPL]), alternative explanations related to structure-specific biodegradation mechanisms or pH-related bioavailability cannot be ruled out.

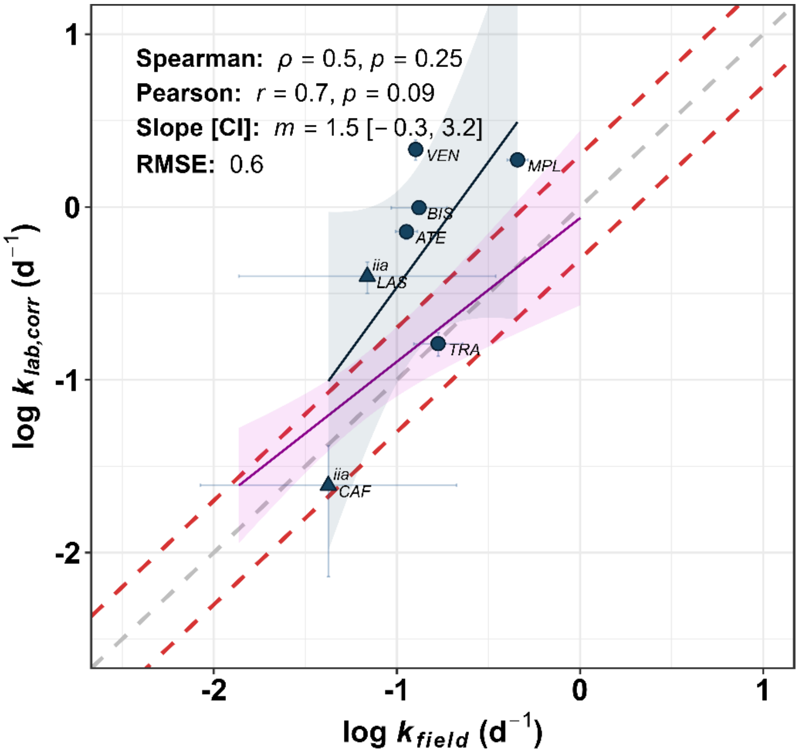

Figure S5: Correlation between log k_{field} and log $k_{lab,corr}$, highlighting the behaviour of strongly sorbing chemicals. Circular markers represent direct injection-derived k_{field}, while triangular markers represent solid-phase-extraction-derived k_{field}. Vertical error bars represent the confidence intervals of the $k_{lab,corr}$ calculation and horizontal error bars represent the error-propagated uncertainty in the k_{field} estimation (95 % confidence intervals). The solid blue line, solid purple line, dashed grey line, and dashed red lines represent the linear regression between $k_{lab,corr}$ and k_{field} for strongly sorbing chemicals, the linear regression between $k_{lab,corr}$ and k_{field} for the remaining 14 chemicals in Figure 2 (excludes outliers), the 1:1 relationship, and the ± factor-of-2 bounds around the 1:1 relationship, respectively. Marker text labels represent both the abbreviated chemical name (Table S1) and the data quality of k_{field}, according to the tiers described in the *Materials and Methods*, where no text label equates to tier (i), i.e., the highest quality. Spearman and Pearson correlation statistics, the slope of the regression and root-mean-square-error (RMSE) for the strongly sorbing subset of chemicals are also displayed.

S7.2 Impact of pH correction

Many of the studied chemicals were ionisable and therefore subject to pH-dependent speciation which can influence the intracellular concentration in degrading microorganisms, and thus the observed rate constant [2]. Since the field pH was ~0.5 log units higher than the laboratory pH, a pH-correction was required in order to compare laboratory and field-derived rate constants. Following methods by Tian *et al.* [2], the laboratory-derived rate constant was multiplied by the fraction neutral quotient (i.e., % neutral species at mean pH_{field} divided by the % neutral species at mean pH_{lab}, see Eqn S4). The neutral fraction (f_N) was predicted using MarvinSketch 23.3 software from ChemAxon.

$$k_{lab} = k_{lab} * \frac{f_{N,\ pH_{field}}}{f_{N,\, pH_{lab}}} \qquad \text{(Eqn S4)}$$

pH correction markedly altered k_{lab} for 12 of the 23 chemicals (≥ 2.5-fold change), with the largest effect for levamisole (LAS), whose k_{lab} increased by almost a factor of 5.

Figure S6 compares log k_{field} and log $k_{lab,corr}$ without pH-correction. The slope of the linear regression deviated further from unity when compared to the pH-corrected data (Figure 2, $m = 0.8$ versus Figure S6, $m = 0.6$), indicating slightly weaker proportional scaling between laboratory and field *k*. The RMSE was also slightly higher for the uncorrected data (0.4 log units as opposed to 0.3 log units for the pH-corrected data). However, even without pH-correction, the data cluster around the 1:1 line, and the same number of chemicals fall within a factor of two of the 1:1 line as in the pH-corrected comparison. This indicates that laboratory and field *k* remain broadly comparable and suggests that biomass densities in the laboratory and field systems were similar. Eight chemicals were flagged as outliers, most of which were weak bases. Based on speciation theory, the lower laboratory pH and therefore reduced neutral fraction (and hence reduced intracellular concentration) of these bases may have reduced their apparent *k*.

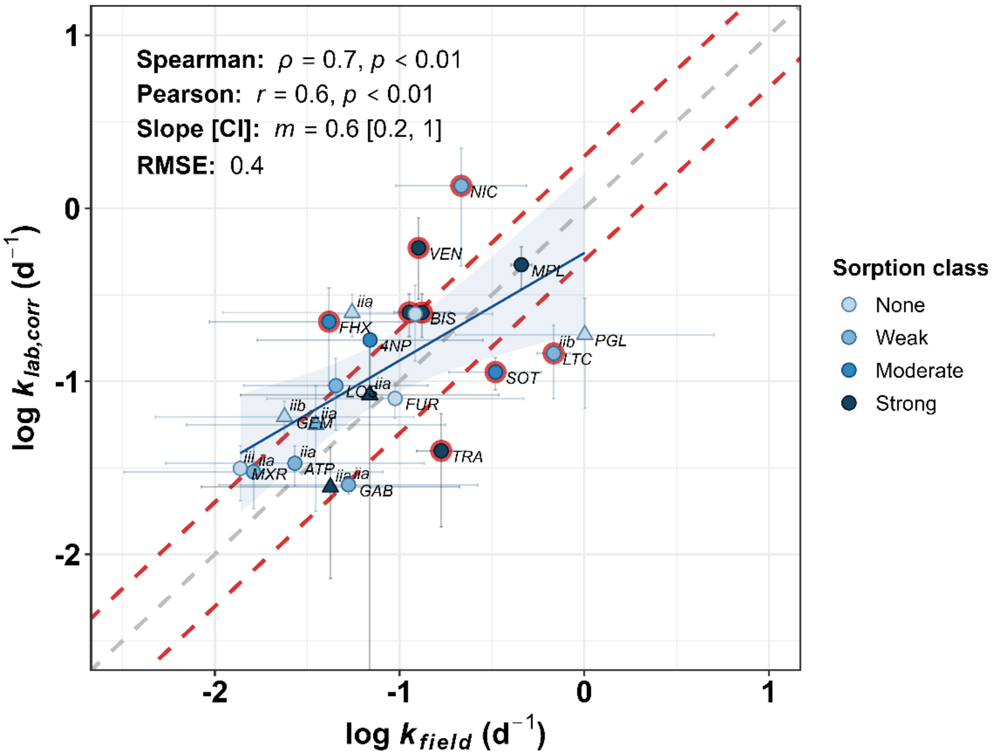

Figure S6: Correlation between k_{field} and $k_{lab,corr}$, where $k_{lab,corr}$ was not corrected for differences in bioavailability owing to pH. Circular markers represent direct injection-derived k_{field}, while triangular markers represent solid-phase-extraction-derived k_{field}. Vertical error bars represent the confidence intervals of the $k_{lab,corr}$ calculation and horizontal error bars represent the error-propagated uncertainty in the k_{field} estimation (95 % confidence intervals). Marker colours represent the different sorption classes (Strong = $f_{dis} < 0.5$, Moderate = $0.5 \geq f_{dis} \leq 0.8$, Weak = $0.8 > f_{dis} < 0.95$, None = $f_{dis} \geq 0.95$). Markers highlighted with red represent outliers (chemicals whose 95 % confidence intervals do not intersect the 1:1 line). The solid blue line, dashed grey line, and dashed red lines, represent the linear regression between $k_{lab,corr}$ and k_{field}, the 1:1 relationship, and the ± factor-of-2 bounds around the 1:1 relationship, respectively. Marker text labels denote the abbreviated chemical name (see Table S1). Spearman and Pearson correlation statistics, the slope of the regression and root-mean-square-error (RMSE) are also displayed.

S7.3 Impact of k_{lab} data choice

In our previous work [20], k_{lab} was derived from data that had been corrected for attenuation occurring in the sterilised sediment control flasks. Due to uncertainty about whether the sterilisation had been completely successful, the data were excluded in those cases where this correction was large (corrected k < 71 % of uncorrected k). In the present study, however, these laboratory data were retained, and the influence of the correction was evaluated as part of the analytical framework, rather than serving as a basis for data exclusion. Recent (unpublished) data indicated that much of the attenuation in the sediment control flasks was due to residual biodegradation. We therefore adopted the uncorrected test flask rate constants as our primary laboratory metric.

The impact of the choice between the uncorrected laboratory-derived rate constant and the value corrected for attenuation occurring in the sterilised sediment control flasks (i.e., k_{test} versus k_{obs}), was evaluated by repeating the comparison of field and laboratory rate constants but using the $k_{lab,corr}$ derived from the k_{obs} dataset (Figure S7). We note that two chemicals (NIC and VEN) were excluded in this comparison due to invalid k_{obs}. The field-to-lab biomass ratio (B_{field}/B_{lab}) for this comparison was 1.1, which was similar to that derived from the uncorrected k_{lab} dataset. Key agreement metrics (Spearman and Pearson correlations, linear regression results, and RMSE) were also similar to those based on the uncorrected k_{lab} dataset, suggesting that our conclusions are robust to k_{lab} dataset choice.

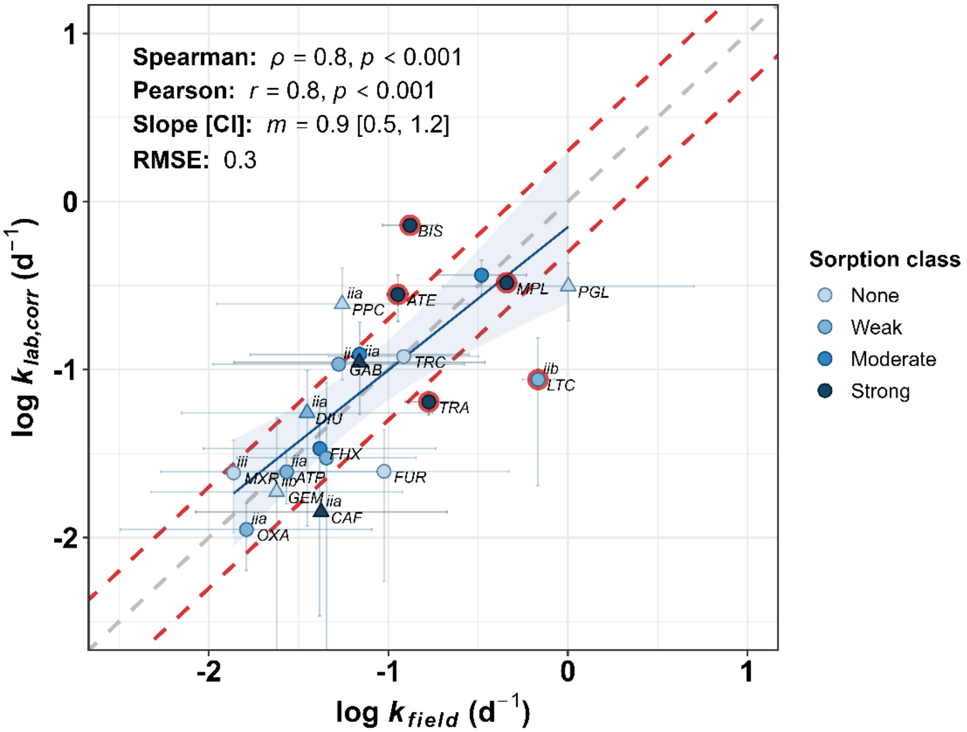

Figure S7: Correlation between k_{field} and $k_{lab,corr}$, where $k_{lab,corr}$ was derived from sediment control corrected k_{lab} data. Circular markers represent direct injection-derived k_{field}, while triangular markers represent solid-phase-extraction-derived k_{field}. Vertical error bars represent the confidence intervals of the $k_{lab,corr}$ calculation and horizontal error bars represent the error-propagated uncertainty in the k_{field} estimation (95 % confidence intervals). Marker colours represent the different sorption classes (Strong = $f_{dis} < 0.5$, Moderate = $0.5 \geq f_{dis} \leq 0.8$, Weak = $0.8 > f_{dis} < 0.95$, None = $f_{dis} \geq 0.95$). Markers highlighted with red represent outliers (chemicals whose 95 % confidence intervals do not intersect the 1:1 line). The solid blue line, dashed grey line, and dashed red lines, represent the linear regression between $k_{lab,corr}$ and k_{field}, the 1:1 relationship, and the ± factor-of-2 bounds around the 1:1 relationship, respectively. Marker text labels represent the data quality of k_{field}, according to the tiers described in the *Materials and Methods*, where no text label equates to tier (i), i.e., the highest quality. Spearman and Pearson correlation statistics, the slope of the regression and root-mean-square-error (RMSE) are also displayed.

S7.4 Impact of photodegradation

Nine chemicals were excluded from the comparison due to suspected photodegradation [23] as shown in Figure S8. Five of these chemicals showed k_{field} > k_{lab} including CAR, KET, SMP, DIC, and CLA. This pattern is consistent with the conceptual expectation that field measurements integrate both biodegradation and photodegradation, whereas the modified OECD 309 experiment was conducted in the dark and therefore isolates (photo-independent) attenuation pathways. In contrast, four chemicals that were also identified as photolabile in the literature (OXP, ABA, ZOL, and STZ) exhibited k_{lab} > k_{field}, despite the absence of light in the laboratory. We cannot attribute this discrepancy to photodegradation, and, given the available data, we refrain from further mechanistic speculation. Instead, we interpret these cases as reminders that, for individual chemicals, differences between k_{field} and k_{lab} may arise from processes other than light exposure (e.g., matrix effects, experimental noise, or uncharacterised environmental drivers) that we are not able to resolve within the scope of this study.

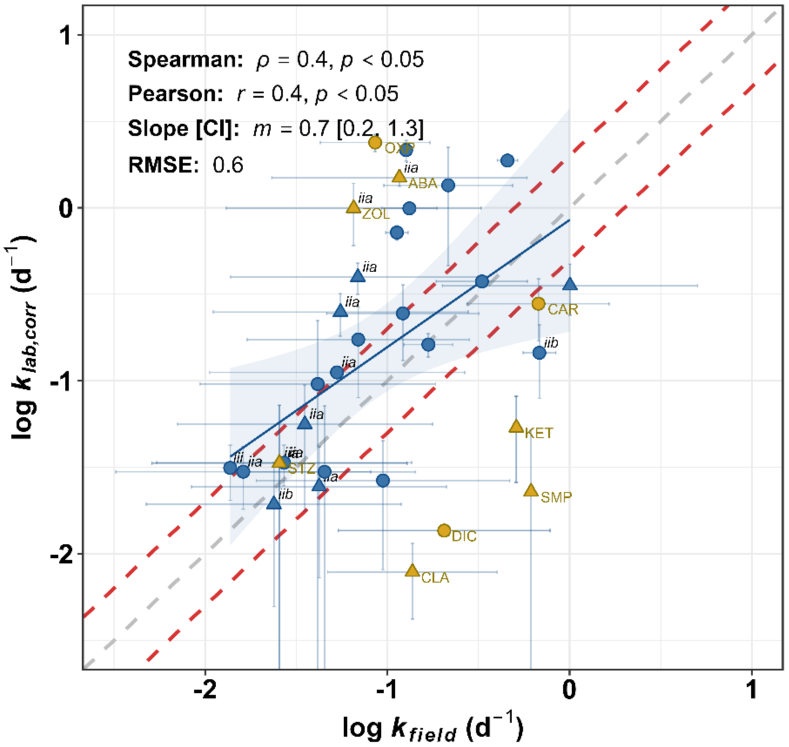

Figure S8: Correlation between k_{field} and $k_{lab,corr}$, where chemicals suspected to photodegrade are included (yellow markers). Circular markers represent direct injection-derived k_{field}, while triangular markers represent solid-phase-extraction-derived k_{field}. Vertical error bars represent the confidence intervals of the $k_{lab,corr}$ calculation and horizontal error bars represent the error-propagated uncertainty in the k_{field} estimation (95 % confidence intervals). The solid blue line, dashed grey line, and dashed red lines, represent the linear regression between $k_{lab,corr}$ and k_{field} (all data), the 1:1 relationship, and the ± factor-of-2 bounds around the 1:1 relationship, respectively. Marker text labels denote the abbreviated chemical name of photodegrading chemicals (see Table S6). Spearman and Pearson correlation statistics, the slope of the regression and root-mean-square-error (RMSE) are also displayed.

Table S6: Summary of photodegradation behaviour for selected chemicals. Results from Paganoni [23] include first-order rate constants for photodegradation (k_p) and biodegradation (k_b), and an indication of whether photodegradation was the dominant loss process (*Photodeg. dominant?*). *Photodegraded? (Literature)* summarises qualitative evidence from other studies, while *Photodegraded?* indicates the final classification adopted in the present study. N.D. = no data.

**Chemical**	**Abbr.**	**Paganoni [23] results**			**Photodegraded? (Literature)**	**Photodegraded?**	**Reference**
		k_p (d^{-1})	k_b (d^{-1})	**Photodeg. Dominant?**			
4-(4-Nitrobenzyl)-pyridine	4NP			N.D.	N.D.	N.D.	
Abacavir	ABA	10.8	1.36	Yes	Yes	Yes	24
Atenolol	ATE			N.D.	No	No	25-29
Acetamiprid	ATP	0.0001	0.0346	No	No	No	30
Bisoprolol	BIS	0.384	0.0703	Yes	No	Uncertain [a]	27,31
Caffeine	CAF			N.D.	Uncertain	Uncertain	29,32-34
Carbendazim	CAR	0.295	0.0118	Yes	Yes	Yes	35-37
Clofibric acid	CLA	0.452	0.915	Yes	Yes	Yes	38-41
Diclofenac	DIC			N.D.	Yes	Yes	21,29,40-44
Diuron	DIU			N.D.	Uncertain	Uncertain	37,44,45
Fenhexamid	FHX			N.D.	Uncertain	Uncertain	46,47
Furosemide	FUR			N.D.	Uncertain	Uncertain	41,48,49
Gabapentin	GAB	0.0001	1.52	No	No	No	44,50,51
Gemfibrozil	GEM			N.D.	Uncertain	Uncertain	29,44,52,53
Ketoprofen	KET	21.1	0.132	Yes	Yes	Yes	29,40,41,44,53
Levamisole	LAS	0.0001	0.035	No	Uncertain	Uncertain	54,55
Losartan	LOS			N.D.	Uncertain	Uncertain	56
Levetiracetam	LTC	0.0001	0.0902	No	N.D.	No	
Metoprolol	MPL	0.24	0.0294	Yes	No	Uncertain	27,29,31
Metoxuron	MXR	0.343	0.0001	Yes	Uncertain	Uncertain [b]	45,57
Nicotinamide	NIC	0.0001	0.961	No	N.D.	No	
Oxazepam	OXA	0.627	0.0001	Yes	Uncertain	Uncertain [b]	58,59
Oxprenolol	OXP	0.957	0.0112	Yes	N.D.	Yes	
Pargyline	PGL	0.101	0.359	Yes	Uncertain	Uncertain [b]	60
Propachlor	PPC	0.0001	0.25	No	Yes	Uncertain [b]	61
Sulfamethoxypyridazine	SMP			N.D.	Yes	Yes	29,62
Sotalol	SOT	0.359	0.0237	Yes	Uncertain	Uncertain [b]	27,31,63
Sulfathiazole	STZ			N.D.	Yes	Yes	64-67
Tramadol	TRA			N.D.	Uncertain	Uncertain [b]	31,68
Triethylcitrate	TRC	0.0001	0.0105	Yes	N.D.	Yes	
Venlafaxine	VEN			N.D.	No	No	31,69-72
Zolpidem	ZOL	12.7	0.248	Yes	Yes	Yes	73

a Paganoni [23] indicated photodegradation ("Yes"), but the wider literature suggested no photodegradation ("No").

b Paganoni [23] indicated photodegradation ("Yes"), but the wider literature was inconclusive ("Uncertain").

S7.5 Evaluation of the theoretical framework and implications for laboratory-to-field extrapolation

Eqn 4 (main text) describes how laboratory-derived biodegradation rate constants can be extrapolated to the field by accounting for differences in biomass density and sediment-water partitioning. For non-sorbing chemicals ($v_S K_{SW} << 1$), Eqn 4 reduces to $k_{field}/k_{lab} \approx B_{field}/B_{lab}$. This provides a practical means of estimating B_{field}/B_{lab} by calculating k_{field}/k_{lab} for non-sorbing reference compounds. A further simplifying assumption in the framework is that active biomass density, like sorption capacity, scales proportionally with the volume fraction of sediment, such that $B_{field}/B_{lab} \approx v_{S,field}/v_{S,lab}$. Under this condition, Eqn 4 predicts that for strongly sorbing chemicals ($v_S K_{SW} >> 1$), the effects of sorption and biomass cancel each other, leading to $k_{lab,corr} \approx k_{lab}$ and hence $k_{field}/k_{lab} \approx 1$. Figure S9 illustrates how these theoretical limits emerge across the transition from dissolved-dominated to sorption-dominated conditions: at low D_{SW} the ratio is controlled by biomass scaling alone, whereas at high D_{SW} it depends on the relative scaling of biomass and sediment volume. When $B_{field}/B_{lab} \approx 1$, deviations from proportional scaling between biomass and sediment would therefore manifest as systematic increases or decreases in k_{field}/k_{lab} for sorbing chemicals, depending on whether biomass density increases or decreases relative to the volume fraction of sorbent (see the green line in Figure S9).

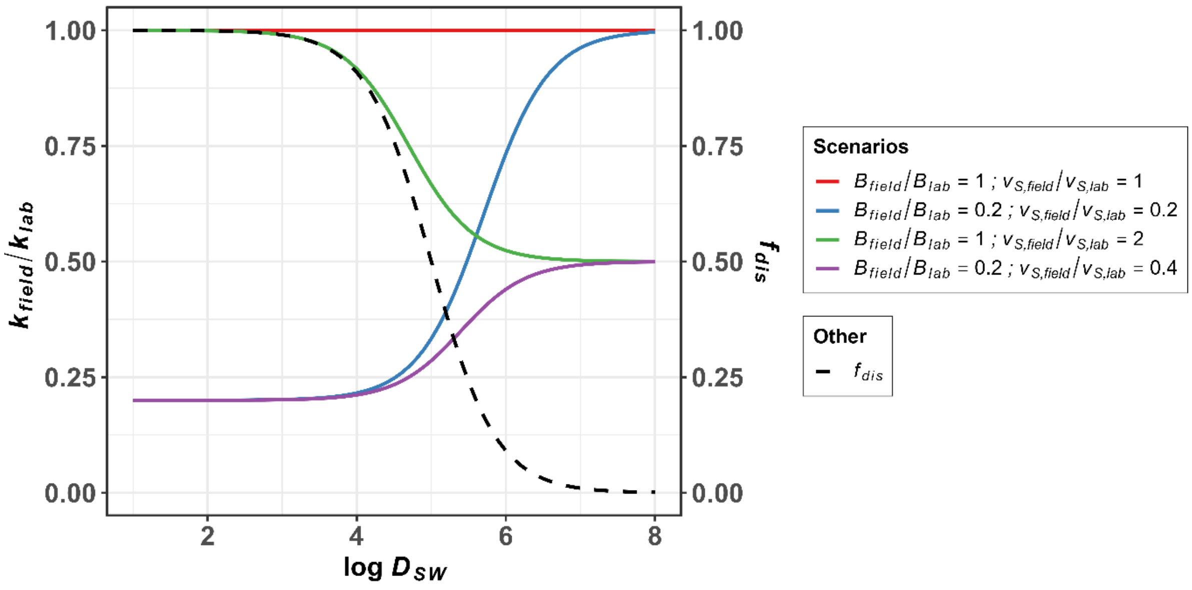

Figure S9: Conceptual evaluation of the influence of sediment-water partitioning on laboratory-to-field biodegradation rate constant extrapolation. The theoretical behaviour of the ratio k_{field}/k_{lab} as a function of the sediment-water distribution ratio (D_{SW}) is depicted under four scenarios. Coloured lines illustrate Eqn 4 evaluated for different combinations of biomass density ratios (B_{field}/B_{lab}) and sediment volume fractions ($v_{S,field}/v_{S,lab}$). The dashed black line shows the corresponding fraction of chemical in the dissolved phase (f_{dis}, secondary y-axis).

References

(1) Tian, R.; Posselt, M.; Miaz, L. T.; Fenner, K.; McLachlan, M. S. Influence of Season on Biodegradation Rates in Rivers. *Environmental Science & Technology* **2024**. DOI 10.1021/acs.est.3c10541.

(2) Tian, R.; Posselt, M.; Fenner, K.; McLachlan, M. S. Variability of Biodegradation Rates of Commercial Chemicals in Rivers in Different Regions of Europe. *Environmental Science & Technology* **2024**, *58* (45), 20201-20210. DOI 10.1021/acs.est.4c07410.

(3) Inland Rail. *Chapter 13 - Surface Water and Hydrology*; Australian Rail Track Corporation Limited, Queensland, **2025**. https://www.statedevelopment.qld.gov.au/coordinator-general/assessments-and-approvals/coordinated-projects/current-projects/inland-rail-border-to-gowrie/inland-rail-border-to-gowrie-projects-eis-documents.

(4) Toowoomba Regional Council. *Toowoomba Region Landscape and Urban Character Study (TRLUCS)*; LAT Studios, Brisbane, Qld, **2025**. https://www.latstudios.com.au/projects/trc-landscape-and-urban-character-study. (accessed 2025/11/25).

(5) Department of Environment, S.; Innovation, Q. *Wetalla Sewage Treatment Plant*. Queensland Government, **2019**. https://wetlandinfo.des.qld.gov.au/wetlands/assessment/monitoring/point-source-release/sewage-treatment-facilities/wetalla/.

(6) Lawrence, M. G.; Bariel, D. G. Tracing treated wastewater in an inland catchment using anthropogenic gadolinium. *Chemosphere* **2010**, *80* (7), 794-799. DOI 10.1016/j.chemosphere.2010.05.001.

(7) Bureau of Meteorology. *Summary Statistics: Toowoomba Airport (Station 041529)*. Australian Government, **2025**. https://www.bom.gov.au/climate/averages/tables/cw_041529.shtml.

(8) Water Monitoring Information Portal Web Service. Queensland Government. https://water-monitoring.information.qld.gov.au/.

(9) Willey, E. C. Urban geology of the Toowoomba conurbation, SE Queensland, Australia. *Quaternary International* **2003**, *103* (1), 57-74. DOI 10.1016/S1040-6182(02)00141-6.

(10) Inland Rail. *Appendix R - Groundwater Technical Report*; Australian Rail Track Corporation Limited, Queensland, **2025**. https://www.statedevelopment.qld.gov.au/coordinator-general/assessments-and-approvals/coordinated-projects/current-projects/inland-rail-border-to-gowrie/inland-rail-border-to-gowrie-projects-eis-documents.

(11) Katz, D. R.; Sullivan, J. C.; Rosa, K.; Gardiner, C. L.; Robuck, A. R.; Lohmann, R.; Kincaid, C.; Cantwell, M. G. Transport and fate of aqueous film forming foam in an urban estuary. *Environmental Pollution* **2022**, *300*, 118963. DOI 10.1016/j.envpol.2022.118963.

(12) Álvarez-Ruiz, R.; Hawker, D. W.; Mueller, J. F.; Gallen, M.; Kaserzon, S.; Picó, Y.; McLachlan, M. S. Postflood Monitoring in a Subtropical Estuary and Benchmarking with PFASs Allows Measurement of Chemical Persistence on the Scale of Months. *Environmental Science & Technology* **2021**, *55* (21), 14607-14616. DOI 10.1021/acs.est.1c02263.

(13) Jaeger, A.; Posselt, M.; Betterle, A.; Schaper, J.; Mechelke, J.; Coll, C.; Lewandowski, J. Spatial and Temporal Variability in Attenuation of Polar Organic Micropollutants in an Urban Lowland Stream. *Environmental Science & Technology* **2019**, *53* (5), 2383-2395. DOI 10.1021/acs.est.8b05488.
(14) Miaz, L. T.; Plassmann, M. M.; Gyllenhammar, I.; Bignert, A.; Sandblom, O.; Lignell, S.; Glynn, A.; Benskin, J. P. Temporal trends of suspect- and target-per/polyfluoroalkyl substances (PFAS), extractable organic fluorine (EOF) and total fluorine (TF) in pooled serum from first-time mothers in Uppsala, Sweden, 1996–2017. *Environmental Science: Processes & Impacts* **2020**, *22* (4), 1071-1083. DOI 10.1039/C9EM00502A.
(15) Engelhardt, J. A.; Plassmann, M. M.; Weiss, J. M. An extended PFAS profiling of a Swedish subpopulation and mixture risk assessments using multiple approaches. *Environment International* **2025**, *195*, 109214. DOI 10.1016/j.envint.2024.109214.
(16) Tian, R.; Posselt, M.; Fenner, K.; McLachlan, M. S. Increasing the Environmental Relevance of Biodegradation Testing by Focusing on Initial Biodegradation Kinetics and Employing Low-Level Spiking. *Environmental Science & Technology Letters* **2023**, *10* (1), 40-45. DOI 10.1021/acs.estlett.2c00811.
(17) Li, Z.; Sobek, A.; Radke, M. Fate of Pharmaceuticals and Their Transformation Products in Four Small European Rivers Receiving Treated Wastewater. *Environmental Science & Technology* **2016**, *50* (11), 5614-5621. DOI 10.1021/acs.est.5b06327.
(18) Li, Z.; Undeman, E.; Papa, E.; McLachlan, M. S. High-throughput evaluation of organic contaminant removal efficiency in a wastewater treatment plant using direct injection UHPLC-Orbitrap-MS/MS. *Environmental Science: Processes & Impacts* **2018**, *20* (3), 561-571. DOI 10.1039/C7EM00552K.
(19) Brunius, C.; Shi, L.; Landberg, R. Large-scale untargeted LC-MS metabolomics data correction using between-batch feature alignment and cluster-based within-batch signal intensity drift correction. *Metabolites* **2020**, *10* (135). DOI 10.3390/metabo10040135.
(20) Weir, L. M.; Tian, R.; Posselt, M.; Mueller, J. F.; McLachlan, M. S. From freshwater to marine environments: spatial variation in chemical biodegradation rates applying a modified OECD 309-type experiment. *Environmental Science & Technology* **2025**. DOI 10.1021/acs.est.5c09998.
(21) Seller, C.; Varga, L.; Börgardts, E.; Vogler, B.; Janssen, E.; Singer, H.; Fenner, K.; Honti, M. Do biotransformation data from laboratory experiments reflect micropollutant degradation in a large river basin? *Water Research* **2023**, *235*, 119908. DOI 10.1016/j.watres.2023.119908.
(22) Tian, R.; Posselt, M.; Miaz, L. T.; Fenner, K.; McLachlan, M. S. Influence of Season on Biodegradation Rates in Rivers. *Environmental Science & Technology* **2024**, *58* (16), 7144-7153. DOI 10.1021/acs.est.3c10541.
(23) Paganoni, L. Relative contribution of photodegradation to the attenuation of trace organic pollutants in rivers. Master's thesis, Stockholm University, **2025**.
(24) Prasse, C.; Wenk, J.; Jasper, J. T.; Ternes, T. A.; Sedlak, D. L. Co-occurrence of Photochemical and Microbiological Transformation Processes in Open-Water Unit Process Wetlands. *Environmental Science & Technology* **2015**, *49* (24), 14136-14145. DOI 10.1021/acs.est.5b03783.
(25) Zeng, C.; Ji, Y.; Zhou, L.; Zhang, Y.; Yang, X. The role of dissolved organic matters in the aquatic photodegradation of atenolol. *Journal of Hazardous Materials* **2012**, *239-240*, 340-347. DOI 10.1016/j.jhazmat.2012.09.005.

(26) Jasper, J. T.; Sedlak, D. L. Phototransformation of Wastewater-Derived Trace Organic Contaminants in Open-Water Unit Process Treatment Wetlands. *Environmental Science & Technology* **2013**, *47* (19), 10781-10790. DOI 10.1021/es304334w.
(27) Piram, A.; Salvador, A.; Verne, C.; Herbreteau, B.; Faure, R. Photolysis of β-blockers in environmental waters. *Chemosphere* **2008**, *73* (8), 1265-1271. DOI 10.1016/j.chemosphere.2008.07.018.
(28) Yamamoto, H.; Nakamura, Y.; Moriguchi, S.; Nakamura, Y.; Honda, Y.; Tamura, I.; Hirata, Y.; Hayashi, A.; Sekizawa, J. Persistence and partitioning of eight selected pharmaceuticals in the aquatic environment: Laboratory photolysis, biodegradation, and sorption experiments. *Water Research* **2009**, *43* (2), 351-362. DOI 10.1016/j.watres.2008.10.039.
(29) Baena-Nogueras, R. M.; González-Mazo, E.; Lara-Martín, P. A. Degradation kinetics of pharmaceuticals and personal care products in surface waters: photolysis vs biodegradation. *Science of the Total Environment* **2017**, *590-591*, 643-654. DOI 10.1016/j.scitotenv.2017.03.015.
(30) Naumann, T.; Bento, C. P. M.; Wittmann, A.; Gandrass, J.; Tang, J.; Zhen, X.; Liu, L.; Ebinghaus, R. Occurrence and ecological risk assessment of neonicotinoids and related insecticides in the Bohai Sea and its surrounding rivers, China. *Water Research* **2022**, *209*, 117912. DOI 10.1016/j.watres.2021.117912.
(31) Schmitt, M.; Wack, K.; Glaser, C.; Wei, R.; Zwiener, C. Separation of Photochemical and Non-Photochemical Diurnal In-Stream Attenuation of Micropollutants. *Environmental Science & Technology* **2021**, *55* (13), 8908-8917. DOI 10.1021/acs.est.1c02116.
(32) Alhassen, H. M.; Dyer, F. J.; Thompson, R. M. The photolytic breakdown of caffeine and paracetamol residues in surface water. *Water Environment Research* **2023**, *95* (8), e10909. DOI 10.1002/wer.10909.
(33) Jacobs, L. E.; Weavers, L. K.; Houtz, E. F.; Chin, Y.-P. Photosensitized degradation of caffeine: Role of fulvic acids and nitrate. *Chemosphere* **2012**, *86* (2), 124-129. DOI 10.1016/j.chemosphere.2011.09.052.
(34) Lam, M. W.; Young, C. J.; Brain, R. A.; Johnson, D. J.; Hanson, M. A.; Wilson, C. J.; Richards, S. M.; Solomon, K. R.; Mabury, S. A. Aquatic persistence of eight pharmaceuticals in a microcosm study. *Environmental Toxicology and Chemistry* **2004**, *23* (6), 1431-1440. DOI 10.1897/03-421.
(35) Yogesh Kumar, K.; Prashanth, M. K.; Parashuram, L.; Palanivel, B.; Alharti, F. A.; Jeon, B.-H.; Raghu, M. S. Gadolinium sesquisulfide anchored N-doped reduced graphene oxide for sensitive detection and degradation of carbendazim. *Chemosphere* **2022**, *296*, 134030. DOI 10.1016/j.chemosphere.2022.134030.
(36) Kaur, T.; Toor, A. P.; Wanchoo, R. K. Parametric study on degradation of fungicide carbendazim in dilute aqueous solutions using nano TiO2. *Desalination and Water Treatment* **2015**, *54* (1), 122-131. DOI 10.1080/19443994.2013.879081.
(37) Li, D.; Ma, X. Y.; Zhang, S.; Wang, Y. K.; Han, Y.; Chen, R.; Wang, X. C.; Ngo, H. H. Aquatic photolysis of high-risk chemicals of emerging concern from secondary effluent mediated by sunlight irradiation for ecological safety and the enhanced methods. *Water Research* **2023**, *238*, 120002. DOI 10.1016/j.watres.2023.120002.
(38) Challis, J. K.; Hanson, M. L.; Friesen, K. J.; Wong, C. S. A critical assessment of the photodegradation of pharmaceuticals in aquatic environments: defining our current

understanding and identifying knowledge gaps. *Environmental Science: Processes & Impacts* **2014**, *16* (4), 672-696. DOI 10.1039/C3EM00615H.
(39) Cardinal, P.; Anderson, J. C.; Carlson, J. C.; Low, J. E.; Challis, J. K.; Beattie, S. A.; Bartel, C. N.; Elliott, A. D.; Montero, O. F.; Lokesh, S.; Favreau, A.; Kozlova, T. A.; Knapp, C. W.; Hanson, M. L.; Wong, C. S. Macrophytes may not contribute significantly to removal of nutrients, pharmaceuticals, and antibiotic resistance in model surface constructed wetlands. *Science of The Total Environment* **2014**, *482-483*, 294-304. DOI 10.1016/j.scitotenv.2014.02.095.
(40) Tixier, C.; Singer, H. P.; Oellers, S.; Müller, S. R. Occurrence and Fate of Carbamazepine, Clofibric Acid, Diclofenac, Ibuprofen, Ketoprofen, and Naproxen in Surface Waters. *Environmental Science & Technology* **2003**, *37* (6), 1061-1068. DOI 10.1021/es025834r.
(41) Hanamoto, S.; Nakada, N.; Yamashita, N.; Tanaka, H. Modeling the Photochemical Attenuation of Down-the-Drain Chemicals during River Transport by Stochastic Methods and Field Measurements of Pharmaceuticals and Personal Care Products. *Environmental Science & Technology* **2013**, *47* (23), 13571-13577. DOI 10.1021/es4035478.
(42) Kanakaraju, D.; Motti, C. A.; Glass, B. D.; Oelgemöller, M. Photolysis and TiO2-catalysed degradation of diclofenac in surface and drinking water using circulating batch photoreactors. *Environmental Chemistry* **2014**, *11* (1), 51-62. DOI 10.1071/EN13098.
(43) Lin, J.; Chi, L.; Yuan, Q.; Li, B.; Feng, M. Photodegradation of typical pharmaceuticals changes toxicity to algae in estuarine water: A metabolomic insight. *Science of The Total Environment* **2024**, *908*, 168338. DOI 10.1016/j.scitotenv.2023.168338.
(44) De la Cruz, N.; Giménez, J.; Esplugas, S.; Grandjean, D.; de Alencastro, L. F.; Pulgarín, C. Degradation of 32 emergent contaminants by UV and neutral photo-fenton in domestic wastewater effluent previously treated by activated sludge. *Water Research* **2012**, *46* (6), 1947-1957. DOI 10.1016/j.watres.2012.01.014.
(45) Fabbri, D.; Minella, M.; Maurino, V.; Minero, C.; Vione, D. Photochemical transformation of phenylurea herbicides in surface waters: A model assessment of persistence, and implications for the possible generation of hazardous intermediates. *Chemosphere* **2015**, *119*, 601-607. DOI 10.1016/j.chemosphere.2014.07.034.
(46) Lambropoulou, D. A.; Konstantinou, I. K.; Albanis, T. A.; Fernández-Alba, A. R. Photocatalytic degradation of the fungicide Fenhexamid in aqueous TiO2 suspensions: Identification of intermediates products and reaction pathways. *Chemosphere* **2011**, *83* (3), 367-378. DOI 10.1016/j.chemosphere.2010.12.006.
(47) National Registration Authority for, A.; Veterinary, C. *Public Release Summary on Evaluation of the New Active Fenhexamid in the Product Teldor 500 SC Fungicide*; National Registration Authority for Agricultural and Veterinary Chemicals, Canberra, Australia, **2001**. https://www.apvma.gov.au/sites/default/files/publication/13706-prs-fenhexamid.pdf.
(48) Sandré, F.; Moilleron, R.; Morin, C.; Garrigue-Antar, L. Comprehensive analysis of a widely pharmaceutical, furosemide, and its degradation products in aquatic systems: Occurrence, fate, and ecotoxicity. *Environmental Pollution* **2024**, *348*, 123799. DOI 10.1016/j.envpol.2024.123799.
(49) Lee, D.; Alyami, I.; Zimila, H.; Arnold, R. G.; Quanrud, D. M.; Sáez, A. E. Photolytic transformation of trace organic compounds: Roles of direct photolysis and indirect photolysis by singlet oxygen. *Water Research* **2025**, *283*, 123799. DOI 10.1016/j.watres.2025.123799.

(50) Neamţu, M.; Grandjean, D.; Sienkiewicz, A.; Le Faucheur, S.; Slaveykova, V.; Colmenares, J. J. V.; Pulgarín, C.; de Alencastro, L. F. Degradation of eight relevant micropollutants in different water matrices by neutral photo-Fenton process under UV254 and simulated solar light irradiation – A comparative study. *Applied Catalysis B: Environmental* **2014**, *158-159*, 30-37. DOI 10.1016/j.apcatb.2014.04.001.
(51) Herrmann, M.; Menz, J.; Olsson, O.; Kümmerer, K. Identification of phototransformation products of the antiepileptic drug gabapentin: Biodegradability and initial assessment of toxicity. *Water Research* **2015**, *85*, 11-21. DOI 10.1016/j.watres.2015.08.004.
(52) Zhang, Y.-n.; Zhou, Y.; Qu, J.; Chen, J.; Zhao, J.; Lu, Y.; Li, C.; Xie, Q.; Peijnenburg, W. J. G. M. Unveiling the important roles of coexisting contaminants on photochemical transformations of pharmaceuticals: Fibrate drugs as a case study. *Journal of Hazardous Materials* **2018**, *358*, 216-221. DOI 10.1016/j.jhazmat.2018.06.068.
(53) Lin, A. Y.-C.; Reinhard, M. Photodegradation of common environmental pharmaceuticals and estrogens in river water. *Environmental Toxicology and Chemistry* **2005**, *24* (6), 1303-1309. DOI 10.1897/04-236R.1.
(54) Yoshimura, H.; Endoh, Y. S. Acute toxicity to freshwater organisms of antiparasitic drugs for veterinary use. *Environmental Toxicology* **2005**, *20* (1), 60-66. DOI 10.1002/tox.20078.
(55) Dolar, D.; Pelko, S.; Košutić, K.; Horvat, A. J. M. Removal of anthelmintic drugs and their photodegradation products from water with RO/NF membranes. *Process Safety and Environmental Protection* **2012**, *90* (2), 147-152. DOI 10.1016/j.psep.2011.08.007.
(56) Ioannidi, A.; Arvaniti, O. S.; Nika, M.-C.; Aalizadeh, R.; Thomaidis, N. S.; Mantzavinos, D.; Frontistis, Z. Removal of drug losartan in environmental aquatic matrices by heat-activated persulfate: Kinetics, transformation products and synergistic effects. *Chemosphere* **2022**, *287*, 131952. DOI 10.1016/j.chemosphere.2021.131952.
(57) Wu, B.; Wang, J.; He, X.; Dai, H.; Zheng, X.; Ma, J.; Yao, Y.; Liu, D.; Yu, W.; Chen, B.; Chu, C. Accelerated Indirect Photodegradation of Organic Pollutants at the Soil–Water Interface. *Environmental Science & Technology* **2024**, *58* (45), 20181-20189. DOI 10.1021/acs.est.4c06993.
(58) Calisto, V.; Domingues, M. R. M.; Esteves, V. I. Photodegradation of psychiatric pharmaceuticals in aquatic environments – Kinetics and photodegradation products. *Water Research* **2011**, *45* (18), 6097-6106. DOI 10.1016/j.watres.2011.09.008.
(59) West, C. E.; Rowland, S. J. Aqueous Phototransformation of Diazepam and Related Human Metabolites under Simulated Sunlight. *Environmental Science & Technology* **2012**, *46* (9), 4749-4756. DOI 10.1021/es203529z.
(60) Gulde, R.; Meier, U.; Schymanski, E. L.; Kohler, H.-P. E.; Helbling, D. E.; Derrer, S.; Rentsch, D.; Fenner, K. Systematic Exploration of Biotransformation Reactions of Amine-Containing Micropollutants in Activated Sludge. *Environmental Science & Technology* **2016**, *50* (6), 2908-2920. DOI 10.1021/acs.est.5b05186.
(61) Konstantinou, I. K.; Zarkadis, A. K.; Albanis, T. A. Photodegradation of Selected Herbicides in Various Natural Waters and Soils under Environmental Conditions. *Journal of Environmental Quality* **2001**, *30* (1), 121-130. DOI 10.2134/jeq2001.301121x.
(62) Khaleel, N. D. H.; Mahmoud, W. M. M.; Hadad, G. M.; Abdel-Salam, R. A.; Kümmerer, K. Photolysis of sulfamethoxypyridazine in various aqueous media: Aerobic biodegradation and identification of photoproducts by LC-UV–MS/MS. *Journal of Hazardous Materials* **2013**, *244-245*, 654-661. DOI 10.1016/j.jhazmat.2012.10.059.

(63) Leresche, F.; von Gunten, U.; Canonica, S. Probing the Photosensitizing and Inhibitory Effects of Dissolved Organic Matter by Using N,N-dimethyl-4-cyanoaniline (DMABN). *Environmental Science & Technology* **2016**, *50* (20), 10997-11007. DOI 10.1021/acs.est.6b02868.
(64) Tang, X.; Cui, Z.; Bai, Y.; Su, R. Indirect photodegradation of sulfathiazole and sulfamerazine: Influence of the CDOM components and seawater factors (salinity, pH, nitrate and bicarbonate). *Science of The Total Environment* **2021**, *750*, 141762. DOI 10.1016/j.scitotenv.2020.141762.
(65) Batista, A. P. S.; Teixeira, A. C. S. C.; Cooper, W. J.; Cottrell, B. A. Correlating the chemical and spectroscopic characteristics of natural organic matter with the photodegradation of sulfamerazine. *Water Research* **2016**, *93*, 20-29. DOI 10.1016/j.watres.2015.11.036.
(66) Niu, X.-Z.; Glady-Croué, J.; Croué, J.-P. Photodegradation of sulfathiazole under simulated sunlight: Kinetics, photo-induced structural rearrangement, and antimicrobial activities of photoproducts. *Water Research* **2017**, *124*, 576-583. DOI 10.1016/j.watres.2017.08.019.
(67) Boreen, A. L.; Arnold, W. A.; McNeill, K. Photochemical Fate of Sulfa Drugs in the Aquatic Environment: Sulfa Drugs Containing Five-Membered Heterocyclic Groups. *Environmental Science & Technology* **2004**, *38* (14), 3933-3940. DOI 10.1021/es0353053.
(68) Glaser, C.; Zarfl, C.; Werneburg, M.; Böckmann, M.; Zwiener, C.; Schwientek, M. Temporal and spatial variable in-stream attenuation of selected pharmaceuticals. *Science of The Total Environment* **2020**, *741*, 139514. DOI 10.1016/j.scitotenv.2020.139514.
(69) Guillet, G.; Knapp, J. L. A.; Merel, S.; Cirpka, O. A.; Grathwohl, P.; Zwiener, C.; Schwientek, M. Fate of wastewater contaminants in rivers: Using conservative-tracer based transfer functions to assess reactive transport. *Science of The Total Environment* **2019**, *656*, 1250-1260. DOI 10.1016/j.scitotenv.2018.11.379.
(70) Li, Z.; Gomez, E.; Fenet, H.; Chiron, S. Chiral signature of venlafaxine as a marker of biological attenuation processes. *Chemosphere* **2013**, *90* (6), 1933-1938. DOI 10.1016/j.chemosphere.2012.10.033.
(71) Rühmland, S.; Wick, A.; Ternes, T. A.; Barjenbruch, M. Fate of pharmaceuticals in a subsurface flow constructed wetland and two ponds. *Ecological Engineering* **2015**, *80*, 125-139. DOI 10.1016/j.ecoleng.2015.01.036.
(72) Writer, J. H.; Antweiler, R. C.; Ferrer, I.; Ryan, J. N.; Thurman, E. M. In-Stream Attenuation of Neuro-Active Pharmaceuticals and Their Metabolites. *Environmental Science & Technology* **2013**, *47* (17), 9781-9790. DOI 10.1021/es402158t.
(73) Malesevic, M.; Zivanovic, L.; Protic, A.; Radisic, M.; Lausevic, M.; Jovic, Z.; Zecevic, M. Stress degradation studies on zolpidem tartrate using LC-DAD and LC-MS methods. *Acta Chromatographica* **2014**, *26* (1), 81-96. DOI 10.1556/achrom.26.2014.1.8.
